# Intraoperative Hypotension Is Associated with Postoperative Nausea and Vomiting in the PACU: A Retrospective Database Analysis

**DOI:** 10.3390/jcm12052009

**Published:** 2023-03-03

**Authors:** Mathias Maleczek, Daniel Laxar, Angelika Geroldinger, Oliver Kimberger

**Affiliations:** 1 Department of Anaesthesiology, Intensive Care Medicine and Pain Medicine, Medical University of Vienna, 1090 Vienna, Austria; 2 Ludwig Boltzmann Institute for Digital Health and Patient Safety, Medical University of Vienna, 1180 Vienna, Austria; 3 Center for Medical Statistics, Informatics and Intelligent Systems, Institute of Clinical Biometrics, Medical University of Vienna, 1090 Vienna, Austria

**Keywords:** PONV, intraoperative hypotension, data science

## Abstract

Multiple risk factors for postoperative nausea and vomiting (PONV)—a very distressing and outcome-related complication—have been identified, including female sex, absence of a history of smoking, history of PONV, and postoperative opioid use. Evidence of association of intraoperative hypotension with PONV is contradictory. A retrospective analysis of the perioperative documentation of 38,577 surgeries was conducted. The associations between different characterizations of intraoperative hypotension and PONV in the postoperative care unit (PACU) were investigated. First, the relationship between different characterizations of intraoperative hypotension with regard to PONV in the PACU was investigated. Secondly, the performance of the optimal characterization was assessed in an independent dataset derived via random split. The vast majority of characterizations showed an association of hypotension with the incidence of PONV in the PACU. In a multivariable regression, time with a MAP under 50 mmHg showed the strongest association with PONV in terms of the cross-validated Brier score. The adjusted odds for PONV in the PACU were estimated to be 1.34 times higher (95% CI: 1.33–1.35) when a MAP was under 50 mmHg for at least 1.8 min than when a MAP remained above 50 mmHg. The finding indicates that intraoperative hypotension may yet be another risk factor for PONV and therefore emphasizes the importance of intraoperative blood pressure management not only in patients at risk for cardiovascular complications but also in young and healthy patients at risk of PONV.

## 1. Introduction

Postoperative nausea and vomiting (PONV) are very distressing for patients [1]. PONV has an incidence of 30% [2] in the general population and 80% in high risk populations [3]. Known risk factors include female sex, use of volatile anesthesia, a history of previous PONV or motion sickness, non-smoker, lower age, postoperative opioid use, and particular types of surgery [3,4].

Preventing PONV may be facilitated by proper risk stratification using scoring systems such as the Apfel score, which includes the factors of female sex, non-smoker, history of PONV or motion sickness, and postoperative opioid use [3]. The score indicates whether PONV prophylaxis should be administered and anesthesia with a lower risk of PONV be performed, i.e., total intravenous anesthesia [5,6,7].

Over the past years, associations between intraoperative hypotension (IOH) and outcomes such as postoperative kidney function, stroke, and myocardial injury have been shown [8,9,10,11]. Furthermore, a “hypotension-dose”-dependent increase in myocardial injury in non-cardiac surgery (MINS) and acute kidney injury (AKI) has been demonstrated [11].

Characterizing intraoperative hypotension is difficult with many different approaches found in the recent literature: [12] Intraoperative hypotension can be defined either by absolute or relative time with a MAP under a certain value or by change from baseline in percent [8,10,11,13]. Another option used in the literature is change from patients’ preoperative blood pressure values [12]. Choosing different blood pressure thresholds potentially changes resulting outcomes and the association of one characterization of hypotension with one detrimental outcome, such as MINS, is not necessarily generalizable for other outcomes, such as PONV.

Although often seen in clinical practice, only a few small trials investigated the influence of intraoperative hypotension on PONV: Pusch et al. examined the effect of systolic arterial pressure variation on PONV in 300 patients and showed that a decrease in systolic blood pressure was associated with an increased incidence of PONV [14]. Heidari et al. showed that preoperative volume loading had an effect on both the blood pressure variability and PONV [15]. Recently, an association between hypotension and PONV was reported in thyroidectomies in 247 patients [16]. Hypoperfusion of the brainstem, as well as intestinal hypoperfusion, was hypothesized as possible mechanisms for PONV promotion in hypotensive patients [14,15,17].

We present the first broad-scale, single-center, retrospective cohort study of a large perioperative database to explore the association of hypotension and PONV. In the present study, we investigated the associations of different features of perioperative hypotension with PONV and tested the best characterization subsequently in an independent dataset.

## 2. Materials and Methods

### 2.1. Study Design

We conducted a retrospective cohort study using the Medical University of Vienna’s perioperative database: Included were all adults (≥18 years) undergoing non-cardiothoracic surgery between 1 September 2013 to 1 September 2020, with a complete record of intraoperative blood pressure, records of PONV in the PACU, and complete data about risk factors of PONV in their perioperative documentation. Risk factors for PONV are collected routinely in the preoperative assessment. Records of PONV in the PACU are documented in the electronic patient data management system. Only patients receiving general anesthesia were included.

### 2.2. Ethics

The University of Vienna’s ethical committee (Borschkegasse 8b/6, 1090 Vienna, President: Prof. Zezula) approved this study and waived the need for informed consent on 21.10.2021 (EKNr: 2062/2020). All research was performed in accordance with relevant guidelines/regulations, especially in accordance with the Declaration of Helsinki.

### 2.3. Data Sources

Data were extracted from the perioperative database and the hospital’s research database. The perioperative research database is constantly synchronized with the Philips IntelliSpace Critical Care and Anesthesia (Philips, Amsterdam, The Netherlands) patient data management system, recording all patient data perioperatively including the ICU. The database contains data for both vital parameters and manually entered observations/actions by all health care professionals. Since 2017, vital parameters have been stored with a resolution of 15 sec; prior to this date the resolution was 2 min.

The second database used is the Medical University of Vienna’s research database: Most of its data are transferred from the hospital information system, an SAP-based (SAP, Walldorf, Germany) database consisting of all patient data except for perioperative and intensive care data. The database includes patient reports, diagnostic codes (International Classification of Diseases, 10th edition), laboratory results and billing information.

### 2.4. Data Processing

Non-invasive blood pressure data were available every time a measurement was initiated (typically every three to five minutes), and invasive blood pressure values every 15 sec or 2 min (as explained above). PONV was documented at the bedside by the nursing staff in either the post anesthesia care unit (PACU) or the intensive care unit (ICU) using four categorical variables: “None”, “Nausea”, “Need to vomit”, “Vomiting”. The first 48 h of ICU and total PACU stay were combined. For analysis purposes, this variable was converted into a dichotomous variable (PONV or No PONV) by aggregating the categories “Nausea”, “Need to vomit” and “Vomiting”. In case of multiple entries, the most severe entity was used: A documentation of “No PONV” was ignored if the entry also contained a documentation of “PONV” as described above. All PONV entries in the first 48 h after end of surgery were included to eliminate other factors such as intensive care therapy.

Artifact removal of blood pressure data was conducted using the following artifact classifications: [11] (1) Systolic pressure greater than or equal to 300 mmHg, (2) Systolic pressure lower than or equal to 20 mmHg, (3) Diastolic pressure lower than or equal to 5 mmHg, (4) Diastolic pressure greater than or equal to 225 mmHg, (5) Systolic pressure lower than or equal to diastolic pressure + 5 mmHg, (6) Values lying outside three standard deviations of each patient’s mean blood pressure. Afterwards, blood pressure values were interpolated linearly, to 15 s intervals where necessary. To assess patients’ comorbidities, the weighted Charlson comorbidity index [18] was calculated using R [19] (R Foundation for Statistical Computing, Vienna, Austria, comorbidity package 0.5.3).

The primary outcome was the occurrence of PONV in the PACU as documented by the staff. The exposure to IOH was a priori defined by thresholds following research on other postoperative outcomes [8,10,20]. The following MAP characterizations were calculated: (1) absolute measures of IOH: (a) Lowest MAP for 1, 3, 5 and 10 sustained minutes [11], (b) Lowest MAP for 1, 3, 5, and 10 cumulative minutes per patient [11], (c) Absolute time with a MAP under 50, 55, 60, 65, 70, 75, or 80 mmHg [11], and (2) Relative time with a MAP under 50, 55, 60, 65, 70, 75, or 80 mmHg compared to duration of anesthesia. Particular MAP value for a certain duration of a hypotensive episode (1, 3, 5, or 10 min) was defined as the upper “low MAP” limit of the lowest MAP episode for the respective number of minutes.

### 2.5. Statistical Analysis

The aims of the study were twofold: (1) To determine which, if any, characterization of hypotension has the strongest association with the occurrence of PONV, and (2) To estimate the association between hypotension (in terms of the characterization that could be identified in the first step) and PONV. The data on the surgical procedures were divided equally into a “shaping dataset” (for the first aim) and an “estimation dataset” (for the second aim) by randomly allocating each patient to either the shaping or the estimation dataset and including all their surgical procedures in the respective dataset. For the first aim, 23 multivariable, generalized, estimating equation logistic regression models with patient as clustering variable were fitted on the “shaping dataset” using an exchangeable correlation structure. Each of the models included one of the characterizations of hypotension and the following adjustment variables: Charlson comorbidity score >0 (yes/no), type of surgery carrying high risk for PONV according to current guidelines [4] (yes/no), smoking status (yes/no), sex (female/male), use of volatile anesthetics (yes/no), use of ondansetron as PONV prophylaxis (yes/no), use of dexamethasone as prophylaxis (yes/no), ASA (four categories), duration of surgery, age and the interaction of age and sex as independent variables and PONV in the PACU as dependent binary variable. Postoperative opioid use was not used as confounder due to its nature as post-exposure covariate, as our model only uses demographic and intraoperative variables [21]. The interaction of age and sex was used after initial analysis of the cohort showed a non-equal distribution of age between male and female patients. Characterizations of hypotension, age, and duration of surgery were represented by natural cubic spline bases using three degrees of freedom. Since the characterizations of hypotension “absolute time with a MAP under 50, 55, 60, 65, 70, 75, or 80 mmHg” and “relative time with a MAP under 50, 55, 60, 65, 70, 75, or 80 mmHg” normalized to duration of anesthesia led to many observations equal to zero, an additional, binary variable indicating whether the value of the characterization of hypotension was equal to zero was included. Characterizations of hypotension were winsorized at the 99th percentile. For each of these multivariable models, the performance was assessed by calculating the Brier score, the discrimination slope, the c-statistic, and the calibration slope, using 10-fold cross-validation with 40 repetitions. In the cross-validation, multiple surgical procedures on one patient were allocated to the same fold. The “best-fitting” characterization of hypotension was determined as the one included in the model with the smallest cross-validated Brier score. In addition to the multivariable logistic regression models, the univariate association between hypotension (in terms of the 23 characterizations represented by natural cubic spline bases) and PONV was investigated.

For the second step, the association between hypotension and PONV in the PACU was estimated by fitting a generalized, estimating equation logistic regression model on the “estimation dataset” with the best-fitting characterization of hypotension identified in step 1 and the adjustment variables (parametrized as in step 1) as independent variable and PONV as dependent variable. The independence of the shaping and the estimation dataset ensures that the estimation of the association between the best-fitting characterization and PONV in the PACU is not affected by multiple comparison bias. Finally, the contribution of the best-fitting characterization was compared against the contribution of the adjustment variables included in the multivariable model by calculating the increase in cross-validated Brier score when excluding one of the variables from the model.

All data processing and statistical calculations were carried out using Python 3.* (primarily with pandas and NumPy packages); (Centrum voor Wiskunde en Informatica, Amsterdam, The Netherlands) and R 4.1.1 (R Foundation for Statistical Computing, Vienna, Austria) [22,23,24].

## 3. Results

Application of the inclusion criteria resulted in 38,577 surgeries on 28,262 patients being included in the analysis before splitting the dataset in two datasets of 19,288 and 19,289 procedures. Overall, 54.5% of surgical procedures were performed on females. The mean age was 53 years. Most procedures were in the field of urology/gynecology/general surgery (42%), followed by orthopedic/trauma surgery (25%) and maxillofacial/ENT surgery (24%). Patients experienced PONV in the PACU in 12.4% (4787) of all included procedures. As expected, Apfel scores differed significantly between the PONV and no PONV group (1.9 vs. 2.4). Primarily Ondansetron and Dexamethasone were used for PONV prophylaxis. Patients at risk of PONV were well detected intraoperatively as those received significantly more Ondansetron (1.8 vs. 1.4 mg, *p* < 0.001) and Dexamethasone (1.8 vs. 2.5 mg, *p* < 0.001) than those without PONV in the PACU. In the complete cohort, 2154 (5.6%) patients were admitted to the ICU; the rest was treated in the PACU. Mean length of stay (LOS) in the PACU was 2.6 h (SD: 3.0), while in the ICU mean LOS was 4.5 days (SD: 11.8 days). The occurrence of PONV was documented on event, and the absence was retrospectively documented in general every 2 h. Patients in PACU were in the nursing staffs’ sight all the time and were thus continuously monitored for PONV episodes. There was a significant difference in total fluid intake (crystalloid and colloid) with patients suffering from PONV receiving 448 mL more (*t*-test: *p* < 0.001). Additional demographics are described in Table 1.

Overall, 6 × 10^6^ individual MAP values were used to calculate the characterizations of MAP with a mean of 79 mmHg and a standard deviation of 1.7 mmHg. Descriptive statistics for all MAP characterizations are found in Table 2. Median lowest MAP for 5 min was 66.0 (61.36–72.00) with a median absolute time with a MAP under 65 mmHg of 6 min.

**Figure 1:**
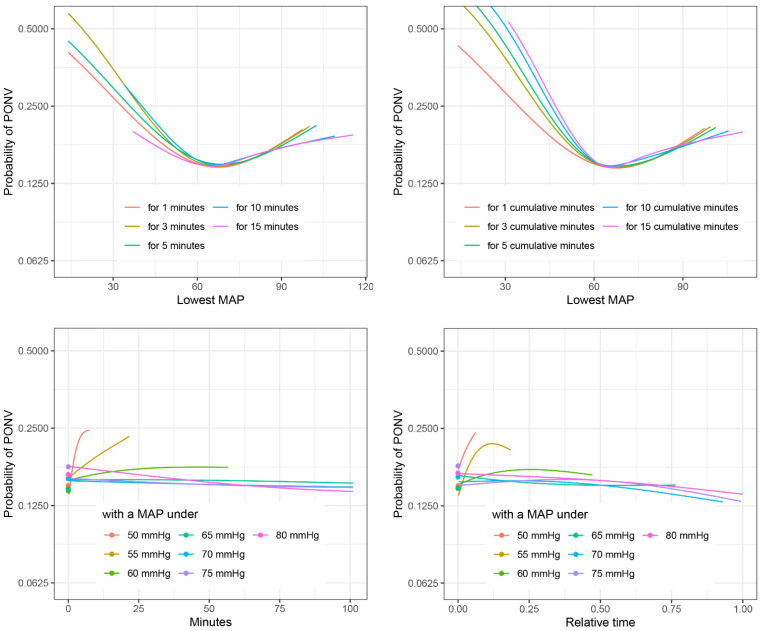
Comparison of different characterizations of PONV. Probability of PONV against different characterizations of IOH for non-smoking females of age 54 with ASA = 2, comorbidity score = 0, use of volatile anesthetics, no use of ondansetron or dexamethasone, and duration of surgery = 1.7 h, estimated in multivariable logistic regression on the shaping dataset. See the Supplementary Materials for similar plots including confidence intervals.

In the shaping dataset, all characterizations of hypotension were significantly associated with PONV in the PACU in univariate analysis (Figure S1). However, in the multivariable logistic regression model, no association could be detected for some characterizations such as minutes with a MAP under 75 mmHg (see Figure 1). When comparing the different characterizations of IOH and their association with PONV, generally a clear rise in risk of PONV could be seen for a MAP < 60 mmHg (Figure 1). Details of all analysis can be found in the Supplementary Materials (Figure S1).

**Figure 2:**
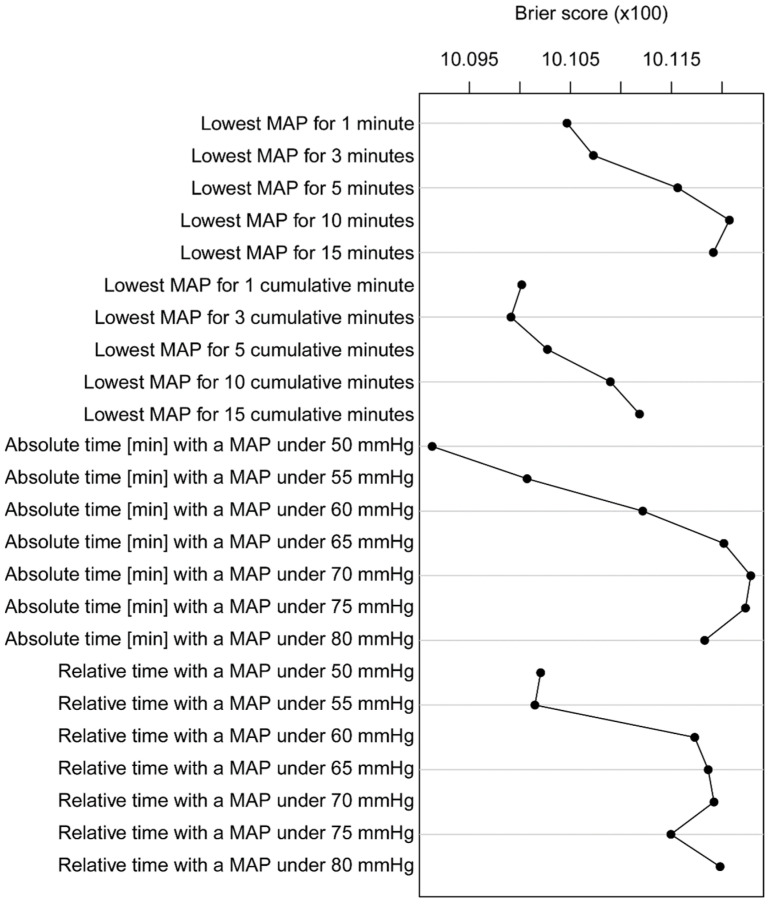
Brier score of different characterizations of PONV. Brier score of the multivariable logistic regression models for all characterizations. The lower the Brier score, the better the performance of the characterization.

Systematic comparison of characterizations in terms of the cross-validated Brier score achieved by the multivariable model showed that the characterization “*time with a MAP < 50 mmHg*” had the strongest association with PONV, followed by *“lowest MAP for 3 cumulative minutes”* and “*lowest MAP for 1 cumulative minute*” (Figure 2).

Using the best-fitting characterization (“*time with a MAP < 50 mmHg*”), the adjusted odds for PONV in the PACU in the independent estimating dataset were estimated to be 1.34 times (95%CI: 1.33–1.35) higher where the MAP was under 50 mmHg for at least 1.8 min than if the MAP was consistently above 50 mmHg (Supplement S1). It is notable that due to only 9.8% of patients experiencing a MAP below 50 mmHg, the confidence interval is relatively broad for non-zero values of the characterization.

For “*time with a MAP <60 mmgHg*”, which reflects a more commonly used characterization in literature, in the shaping dataset the adjusted odds for PONV in the PACU were estimated to be 1.17 times higher (95% CI: 1.16–1.17) when a MAP was under 60 mmHg for at least 6 min than when a MAP remained above 60 mmHg. For comparison, in the shaping dataset, the adjusted odds for PONV in the PACU were estimated to be 1.26 times higher (95% CI: 1.26–1.27) when MAP was under 50 mmHg for at least 1.8 min than when a MAP remained above 50 mmHg. A small difference between the two randomly split datasets was found.

**Figure 3:**
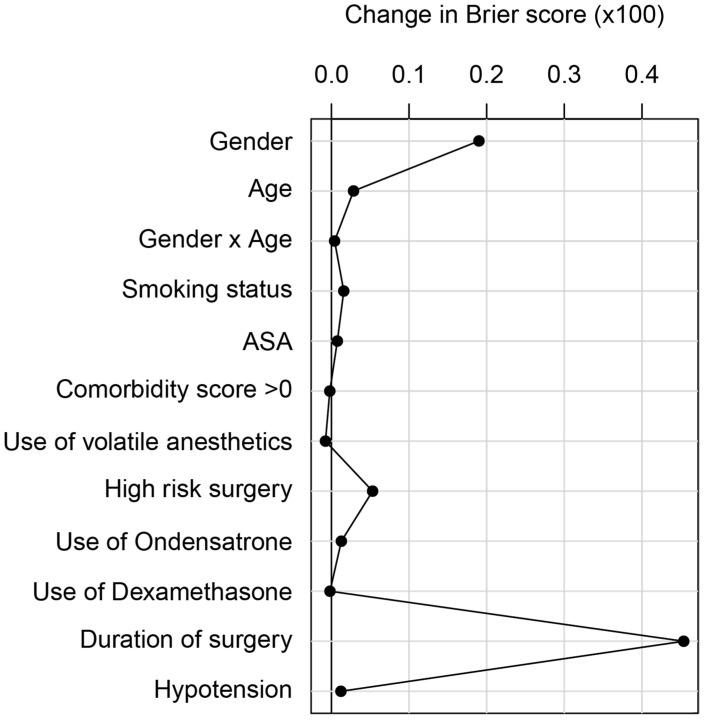
Brier score of different confounders. Relative contribution of the variables in the multivariable model, quantified by the difference in 10-fold cross-validated Brier score (with 40 repetitions) between the model with the respective variable omitted and the full model (described in Figure 3). When evaluating the contribution of sex or age, the interaction of sex and age was also excluded from the model. The larger the difference in Brier score, the higher the contribution of the variable.

Analysis of the influence of the different adjusting variables showed that in the multivariable model the duration of surgery made the most significant contribution in terms of change in cross-validated Brier score (0.005), followed by sex and age (Figure 3).

## 4. Discussion

The association between IOH and PONV has been reported in a small number of earlier trials [14,16,17], but it remains controversial [25]. As PONV is very distressing and has a high incidence in the general surgical population, further reduction and prophylactic strategies are of particular importance. The present analysis showed a distinct association between intraoperative hypotension and PONV in the PACU, with an adjusted odds ratio of 1.34 (95%CI: 1.33–1.35) for a MAP under 50 mmHg for at least 1.8 min versus a MAP consistently above 50 mmHg. As demonstrated in the present study, IOH appears to be a relevant risk factor in addition to well-known factors including female sex, history of PONV, risk surgery, use of volatile anesthesia, non-smoking, duration of surgery, and age [4]. The choice of ”*time with a MAP under 50 mmHg*” as the best fitting characterization of IOH is based on a comparison of various characterizations in the shaping dataset.

The reasons for the association between IOH and PONV may be hypothesized as multifactorial: To date, reduced gut hypoperfusion [26] has been reported as a probable mechanism, as well as orthostatic dysfunction due to hypotension [17]. As PONV is also caused by decreased gastric emptying, which itself is influenced by intestinal perfusion, this could be a contributing factor, although the short duration of IOH necessary to increase PONV contradicts this theory [27]. A known prevention strategy for PONV is supplementation with intravenous fluids, possibly preventing or correcting IOH to some degree [28]. Whether the use of vasopressors to counteract IOH would lead to reduced incidence of PONV remains unclear and needs to be evaluated subsequently.

Undoubtedly, an important risk factor for PONV is postoperative opioid use. However postoperative opioid use was not included in the model, since it comprised intraoperative variables only; thus, postoperative opioid use was invariably a “post-exposure” variable [21]. In contrast, duration of surgery was included in the model, not at least since longer surgeries often require more extensive postoperative pain therapy, which may be one of the reasons for duration of surgery being a promoting factor for PONV. As only a small proportion of patients (5.6%) were admitted to the ICU, this factor was not used as covariable in the model.

Another possible factor would be the route of application of opioids: In the described cohort, standard procedure in the PACU includes bolus application of opioids. In the ICU, opioids are given either via bolus or infusion. PCA usage is mostly used in the wards. PONV prophylaxis in general plays an important role in routine anesthetic care: Typically, guided by scores such as Apfel or Koivuranta, patients at risk are identified and receive a combination of up to four different prophylaxis agents, including 5HT3 receptor antagonists, dexamethasone, antihistamines, dopamine antagonists, and NK-1 receptor antagonists [3,4,27]. Up to now prevention of intraoperative hypotension as a prophylactic precaution was not part of a routine PONV prevention. Further research must prove whether pre-emptive therapy for IOH can serve as additional effective PONV prophylaxis.

The results of the present study are in accordance with a series of recent publications examining the influence of IOH on a variety of postoperative outcomes. First, the association between IOH and myocardial injury was demonstrated, followed by reports of IOH being associated with AKI [8,10,11,20,29]. Outcomes such as delirium, stroke, and overall organ injury have also been shown to be associated with various characterizations of IOH [13,30,31]. Consequently, examining other postoperative outcomes and their association with IOH is of the utmost interest.

Looking at the recent literature clearly indicates a problem with the heterogeneity of definitions of IOH [13]. A wide variety of characterizations is currently being used, ranging from time with a MAP below a certain value to a relative decrease in blood pressure from a patient-derived baseline value [12]. Since it is unlikely that one characterization of IOH is applicable to all postoperative outcomes, our first step was to search for the optimal characterization of IOH to predict PONV. To achieve this, the patient cohort was randomly split in half, with the first set used only to calculate the optimal characterization. The second dataset was used to confirm the association between PONV and this characterization.

Due to incomplete preoperative vital signs, it was not possible to use characterizations applying a decrease from preoperative baseline. However, the current literature suggests that relative changes of blood pressure may be comparably or even less relevant than absolute MAP limits regarding patient outcome [11,32]. As Figure 1 shows, most characterizations analyzed in the present study showed an increased risk of PONV below 60 mmHg, independently of being based on time below a certain MAP or sustained MAP for a certain time.

When comparing these data to the current literature, a cut-off between 60 mmHg and 65 mmHg is found in most of the studies, when absolute characterizations of IOH were used as in the present study [8,11,13]. For sensitivity analysis purposes, the odds ratio of “*time with a MAP <60 mmHg*” was calculated in the shaping dataset and showed very similar results. 

Nevertheless, looking at associations between perioperative hypotension and various patient outcomes, the application and testing of different forms of these characterizations as a first step in comparison to using a single predefined IOH characterization derived from another outcome may be an obvious approach, since a “one-size-fits-all outcome” characterization of perioperative hypotension is unlikely [12].

Another topic that is also still an open research question is the prevention and treatment of IOH. While a variety of treatments for hypotension is well established, and primarily consists of the use of various vasopressors and fluid administration [33], the actual beneficial effect of treating IOH on the reported outcomes remains unclear. As blood pressure is only one part of a set of complex hemodynamic changes during anesthesia, any “optimal” treatment may well be more complex and “personalized” than a general approach [34].

Since the focus of the present analysis was the association of the “net IOH burden” with PONV in the PACU, the inclusion of the numerous possible treatments of IOH (ranging, e.g., from surgical interventions to blood/fluid administration to medications, which may or may not be initiated by the occurrence an IOH episode) would not have elucidated the primary question of the analysis, which is why the authors decided not to include the current multitude of potential IOH therapies. Further research will be necessary to identify the “personalized” treatment option for IOH episodes in individual patients, not at least in order to reduce risk of PONV. Irrespective of the possible treatments, when IOH has already occurred, prediction and prevention may be the best approach—and while the effect on actual patient outcome remains still to be determined, in a first step, some trials have shown the feasibility of predicting and thus preventing IOH [35,36,37]. This approach was mostly successful, but there were also negative results, where blood pressure did not differ between patients with predictive monitoring vs. routine monitoring [33,38,39]. The reasons for this discrepancy vary, ranging from ignoring alarms to improper therapeutic measures. Larger randomized controlled trials focused on outcome and not only on blood pressure are missing to date. Subsequent prospective trials need to be conducted to show potential, personalized IOH treatment options that improve not only blood pressure but also patient outcomes, and to move on from associations to outcome-improving preventive measures.

One possible limitation of this trial is the low overall incidence of PONV—only 12.4% compared to values of up to 30% [2] as reported in the current literature. This may be due to two main causes. Firstly, compared to other studies that also include the first 24–48 h in wards, in this study only information from the PACU and ICU was available. Deducting from the significantly higher doses of intraoperative ondansetron and dexamethasone in the PONV group, a very good risk stratification took place in the described cohort. When looking at PONV in the PACU as this study did, the PONV incidence is quite comparable to the recent literature reporting PONV incidences of 10–20% in cohorts receiving PONV prophylaxis [40,41,42]. As there is no indication that IOH causes only early or delayed PONV, we assumed that while the shorter observational interval does influence total PONV numbers, it does not influence the association between IOH and PONV. The second possible cause is underreporting by PACU staff. This probably happens independently of the patients’ intraoperative blood pressure. Again, such underreporting would only reduce the total number of PONV rather than introduce bias into the estimation of the relationship between IOH and PONV. As we included only patients with a non-empty PONV value in the patient data management system, the risk of patients wrongly being classified as not having PONV is likely to be negligible.

When looking at the usage of PONV prophylaxis in the described cohort, good risk stratification by the treating teams is present: Both intraoperative dexamethasone and ondansetron use was significantly higher in the PONV group.

About 80% of our patients had a non-invasive measurement of their blood pressure at 1 to 5 min intervals. Linear interpolation was used to provide reasonable estimates of intervening values. Obviously, this introduces a small decrease in accuracy compared to invasive measurements available in an interval of up to 15 sec, although it seems unlikely that a further increase in resolution would have changed the characterizations of blood pressure. To ensure this statement, blood pressure characterizations with and without interpolation were compared and resulted in the same measures of central tendency.

The analysis of mean arterial pressure values rather than systolic values was based on two considerations. First, the mean arterial pressure is the value representing organ perfusion best. Second, by technical means, when taking an oscillometric blood pressure, only the MAP is measured directly, while systolic and diastolic pressures are calculated [43].

As this is a retrospective analysis, confounding bias is of concern. We addressed this issue by adjusting for a list of potential confounding variables known from the literature. As with every association study, also in the present analysis an *association* between different characterizations of IOH and PONV was shown, and it is important to bear in mind that this does not necessarily indicate a causal relationship between these two parameters.

As the study’s focus was the development of a prediction model using intraoperative variables for a postoperative prediction of PONV at any time after surgery, “post-exposure” covariates that occurred simultaneously to the outcome of interest were not added to the analysis.

Finally a well-known risk factor for intraoperative hypotension and nausea or vomiting is spinal anesthesia for periparturients [4]. As regional anesthesia was excluded in this trial, there is no risk for introducing bias due to this fact.

When applying the results of the present study in clinical practice, it should be remembered that IOH is associated with PONV, even after short exposure to IOH. Therefore, it could be argued that in patients with high risk of PONV, as well as applying pharmacological PONV prophylaxis, keeping blood pressure always above 60 mmHg may be an important element in the toolbox for PONV prevention.

## 5. Conclusions

A distinct association between intraoperative hypotension and PONV in the PACU could be demonstrated. Intraoperative hypotension may thus be yet another risk factor for PONV, which further emphasizes the significance of preventing intraoperative hypotension to improve a multitude of postoperative outcomes including PONV.

## Figures and Tables

**Table 1:** Shows demographic details of the included procedures.

	All	No PONV	PONV
***n***	38,577	33,790	4787
**Female**	21,037 (54.53%)	17,577 (52.02%)	3460 (72.28%)
**Age, mean (SD)**	52.83 (17.06)	52.89 (17.08)	52.44 (16.90)
**ASA status, n (%)**			
**ASA 1**	18,536 (48.05%)	16,161 (47.83%)	2375 (49.61%)
**ASA 2**	10,073 (26.1%)	8717 (25.80%)	1356 (28.33%)
**ASA 3**	9342 (24.22%)	8381 (24.80%)	961 (20.08%)
**ASA 4**	626 (1.62%)	531 (1.57%)	95 (1.98%)
**Inhalational anesthesia, n (%)**	24,838 (64.39%)	21,435 (63.44%)	3403 (71.09%)
**Smoker** **, n (%)**	12,547 (32.52%)	11,255 (33.31%)	1292 (26.99%)
**History of PONV**, **n (%)**	5902 (15.30%)	4726 (13.99%)	1176 (24.57%)
**Postoperative opioids** **, n (%)**	21,577 (55.93%)	18,001 (53.27%)	3576 (74.70%)
**Risk surgery** **, n (%)**	6836 (17.72%)	5470 (16.19%)	1366 (28.54%)
**Apfel score** **, mean (SD)**	1.93 (1.02)	1.86 (1.01)	2.44 (0.93)
**Dexamethasone intraOP [mg] (SD)**	1.8 (4.2)	1.8 (4.0)	2.5 (5.3)
**Dexamethasone postOP [mg] (SD)**	0.2 (2.4)	0.1 (2.0)	1.0 (4.0)
**Ondansetron intraOP [mg] (SD)**	1.5 (2.3)	1.4 (2.3)	1.8 (2.6)
**Ondansetron postOP [mg] (SD)**	0.8 (2.4)	0.2 (5.1)	1.1 (4.1)
**Fluid intake [ml] (SD)**	1128 (996)	1071 (928)	1519 (1312)
**Push-dose Norepinephrine [mcg] (SD)**	0.6 (107)	0.7 (114.4)	0.06 (2.0)
**Push-dose Phenylephrine [mg] (SD)**	0.1 (0.3)	0.1 (0.3)	0.2 (0.4)
**Norepinephrine infusion (mcg/kg/min) (SD)**	0.003 (0.02)	0.003 (0.02)	0.007 (0.04)
**Surgical area, n (%)**			
**Maxillofacial, ENT, derma** **, n (%)**	9150 (23.72%)	8672 (25.66%)	478 (9.99%)
**Orthopedics, trauma** **, n (%)**	9505 (24.64%)	8806 (26.07%)	699 (14.60%)
**Neurosurgery** **, n (%)**	1635 (4.24%)	923 (2.73%)	712 (14.87%)
**Urology, gynecology, general surgery** **, n (%)**	16,127 (41.80%)	13,469 (39.86%)	2658 (55.52%)
**Other** **, n (%)**	333 (0.86%)	294 (0.87%)	39 (0.81%)
**Non-OR anesthesia, obstetrics** **, n (%)**	793 (2.06%)	728 (2.15%)	65 (1.36%)
**Robotic surgery** **, n (%)**	1034 (2.68%)	898 (2.66%)	136 (2.84%)
**PACU-LOS [h] (SD)**	2.6 (3.0)	2.5 (2.9)	3.5 (3.5)
**ICU-LOS [d] (SD)**	4.5 (11.8)	6.0 (14.7)	2.9 (7.1)
**Charlson comorbidity score, mean (SD)**	0.97 (1.68)	0.93 (1.64)	1.18 (1.92)
**Myocardial infarction** **, n (%)**	110 (0.29%)	85 (0.25%)	25 (0.52%)
**Congestive heart failure** **, n (%)**	316 (0.82%)	277 (0.82%)	39 (0.81%)
**Peripheral vascular disease**, **n (%)**	768 (1.99%)	678 (2.01%)	90 (1.88%)
**Cerebrovascular accident** **, n (%)**	958 (2.48%)	717 (2.12%)	241 (5.03%)
**Dementia** **, n (%)**	50 (0.13%)	42 (0.12%)	8 (0.17%)
**COPD** **, n (%)**	1387 (3.60%)	1209 (3.58%)	178 (3.72%)
**Rheumatic disease** **, n (%)**	169 (0.44%)	140 (0.41%)	29 (0.61%)
**Peptic ulcer disease** **, n (%)**	149 (0.39%)	135 (0.40%)	14 (0.29%)
**Liver disease** **, n (%)**	850 (2.20%)	726 (2.15%)	124 (2.59%)
**Diabetes mellitus** **, n (%)**	1776 (4.60%)	1484 (4.39%)	292 (6.10%)
**Diabetes mellitus with complication** **, n (%)**	441 (1.14%)	412 (1.22%)	29 (0.61%)
**Hemiplegia**	48 (0.12%)	39 (0.12%)	9 (0.19%)
**Renal disease**	1635 (4.24%)	1419 (4.20%)	216 (4.51%)
**Solid tumor**	9167 (23.76%)	7893 (23.36%)	1274 (26.61%)
**Leukemia**	55 (0.14%)	52 (0.15%)	3 (0.06%)
**Metastatic tumor**	1268 (3.29%)	1009 (2.99%)	259 (5.41%)
**AIDS**	60 (0.16%)	60 (0.18%)	0 (0.00%)

ENT: Otorhinolaryngology, LOS: length of stay, intraOP: intraoperatively, postOP: postoperatively, n: number, SD: standard deviation.

**Table 2:** Shows median (first quartile, third quartile) for all characterizations in all surgical procedures, both in the group of procedures free of PONV and in the group of procedures experiencing PONV.

	All *(n* = 38,577)	No PONV (*n* = 33,790)	PONV *(n* = 4787)
**Lowest MAP for**			
1 min	61.08 (56.17, 67.75)	61.45 (56.50, 68.00)	59.00 (54.50, 65.00)
3 min	64.00 (59.25, 69.92)	64.00 (59.50, 70.08)	62.27 (58.00, 67.54)
5 min	66.00 (61.36, 72.00)	66.16 (61.59, 72.00)	64.83 (60.17, 70.00)
10 min	70.00 (65.00, 76.00)	70.00 (65.00, 76.18)	69.00 (64.00, 74.00)
15 min	73.00 (67.75, 79.50)	73.00 (68.00, 80.00)	72.00 (67.00, 77.88)
**Lowest MAP for**			
1 cumulative minute	61.00 (56.00, 67.57)	61.25 (56.20, 68.00)	58.88 (54.00, 64.40)
3 cumulative minutes	63.00 (58.00, 69.12)	63.00 (58.42, 69.64)	60.90 (56.20, 66.22)
5 cumulative minutes	64.00 (59.50, 70.44)	64.50 (59.88, 70.91)	62.00 (58.00, 67.73)
10 cumulative minutes	66.50 (61.70, 73.00)	67.00 (62.00, 73.18)	64.33 (60.00, 70.00)
15 cumulative minutes	68.08 (63.00, 75.00)	68.55 (63.40, 75.20)	66.00 (61.46, 72.00)
**Absolute time [min] with a MAP**			
under 50 mmHg	0.00 (0.00, 0.00)	0.00 (0.00, 0.00)	0.00 (0.00, 0.00)
under 55 mmHg	0.00 (0.00, 0.00)	0.00 (0.00, 0.00)	0.00 (0.00, 1.25)
under 60 mmHg	0.00 (0.00, 5.50)	0.00 (0.00, 5.00)	1.75 (0.00, 9.00)
under 65 mmHg	6.00 (0.00, 21.50)	5.50 (0.00, 20.25)	10.75 (1.00, 30.75)
under 70 mmHg	20.00 (3.75, 47.25)	19.00 (3.25, 45.00)	30.75 (9.25, 66.75)
under 75 mmHg	38.25 (14.75, 74.75)	36.25 (13.75, 70.75)	55.00 (22.75, 111.50)
under 80 mmHg	54.75 (27.00, 98.25)	52.25 (26.00, 92.50)	79.50 (38.00, 150.00)
**Relative time with a MAP**			
under 50 mmHg	0.00 (0.00, 0.00)	0.00 (0.00, 0.00)	0.00 (0.00, 0.00)
under 55 mmHg	0.00 (0.00, 0.00)	0.00 (0.00, 0.00)	0.00 (0.00, 0.00)
under 60 mmHg	0.00 (0.00, 0.04)	0.00 (0.00, 0.04)	0.01 (0.00, 0.05)
under 65 mmHg	0.05 (0.00, 0.19)	0.05 (0.00, 0.19)	0.07 (0.00, 0.18)
under 70 mmHg	0.20 (0.04, 0.43)	0.20 (0.03, 0.44)	0.20 (0.07, 0.41)
under 75 mmHg	0.41 (0.17, 0.67)	0.41 (0.16, 0.67)	0.41 (0.20, 0.64)
under 80 mmHg	0.61 (0.34, 0.82)	0.61 (0.34, 0.82)	0.61 (0.37, 0.81)

## Data Availability

Data and Code are available from the corresponding author upon reasonable request.

## Supplementary Materials

The following supporting information can be downloaded at: https://www.mdpi.com/article/10.3390/jcm12052009/s1, Figure S1: Univariate and multivariate models for all characterizations of IOH and the risk of PONV in the shaping dataset.
