# Supplementary material for: Intraoperative Hypotension Is Associated with Postoperative Nausea and Vomiting in the PACU: A Retrospective Database Analysis
Source: J Clin Med. 2023 Mar 3;12(5):2009. doi: 10.3390/jcm12052009 (PMC10004657; doi:10.3390/jcm12052009)
Supplement: Supplementary file 1 [file jcm-12-02009-s001.zip › jcm-2237631-supplementary.pdf]

Supplement

In this supplement, the univariable and multivariable models of all operationalizations are shown as described in the main text. For all, the probability of PONV against the used operationalization is shown. The gray area marks the pointwise 95% confidence intervals. Note the value and CI of zerovalues.

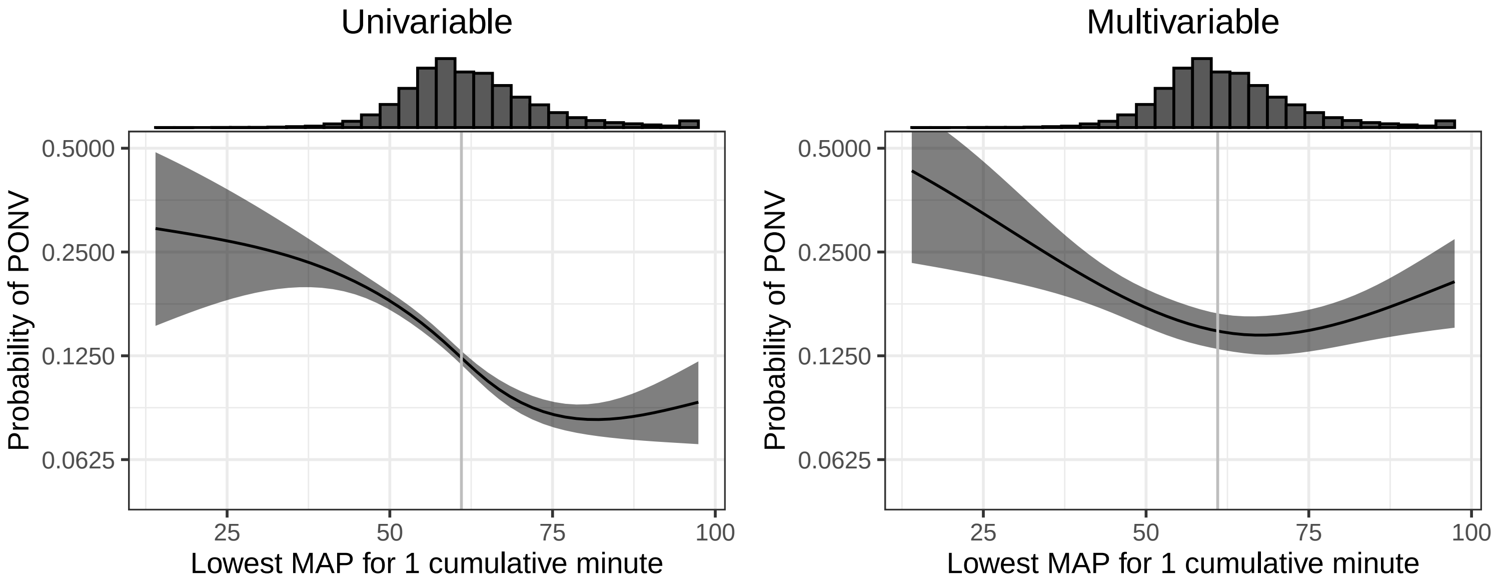

Univariable
Multivariable
Probability of PONV
0.5000
0.2500
0.1250
0.0625
25
50
75
100
Lowest MAP for 1 cumulative minute

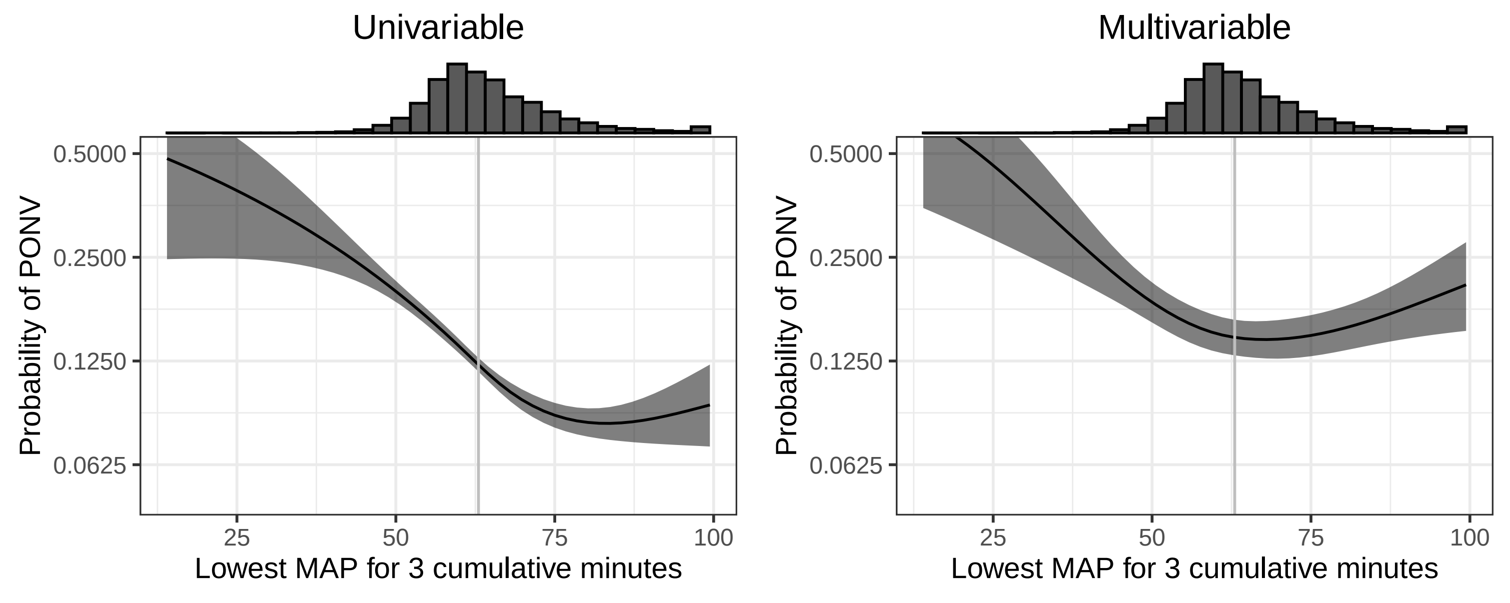

Univariable
Multivariable
Probability of PONV
0.5000
0.2500
0.1250
0.0625
25
50
75
100
Lowest MAP for 3 cumulative minutes

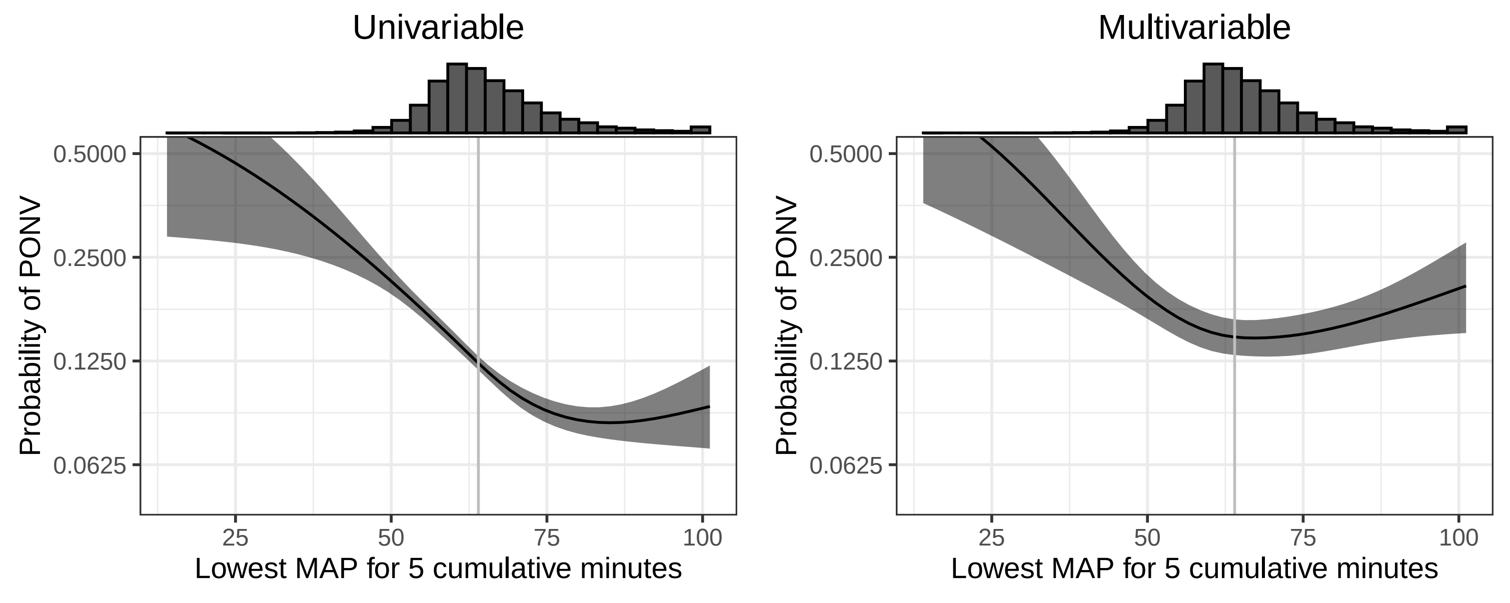
Univariable
Multivariable
Probability of PONV
0.5000
0.2500
0.1250
0.0625
25
50
75
100
Lowest MAP for 5 cumulative minutes

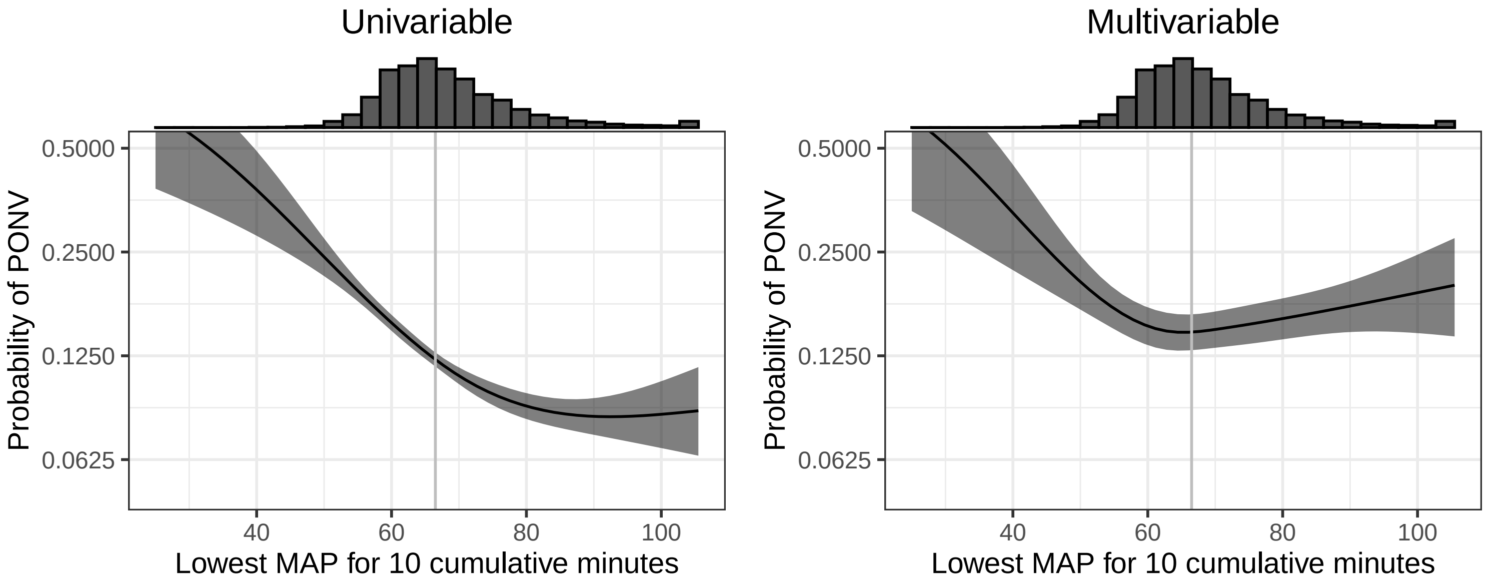

Univariable
Multivariable
0.5000
0.2500
0.1250
0.0625
Probability of PONV
40
60
80
100
Lowest MAP for 10 cumulative minutes

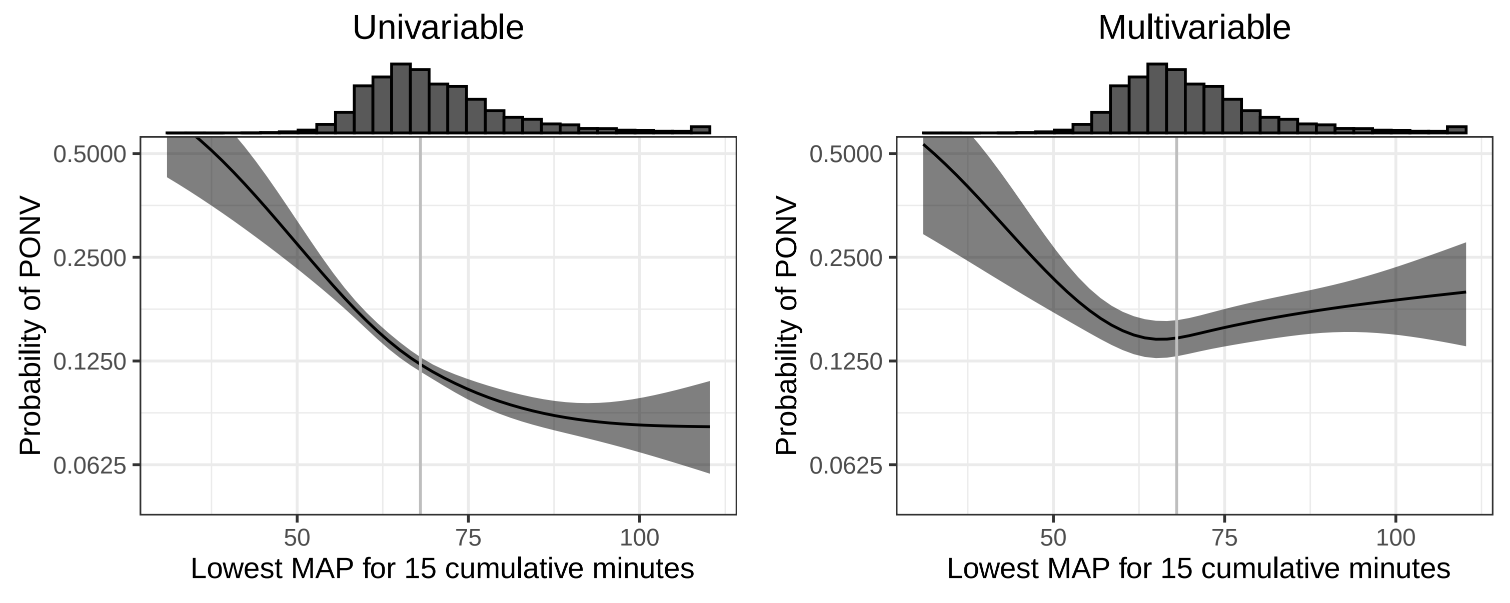
Univariable
Multivariable
Probability of PONV
0.5000
0.2500
0.1250
0.0625
50
75
100
Lowest MAP for 15 cumulative minutes

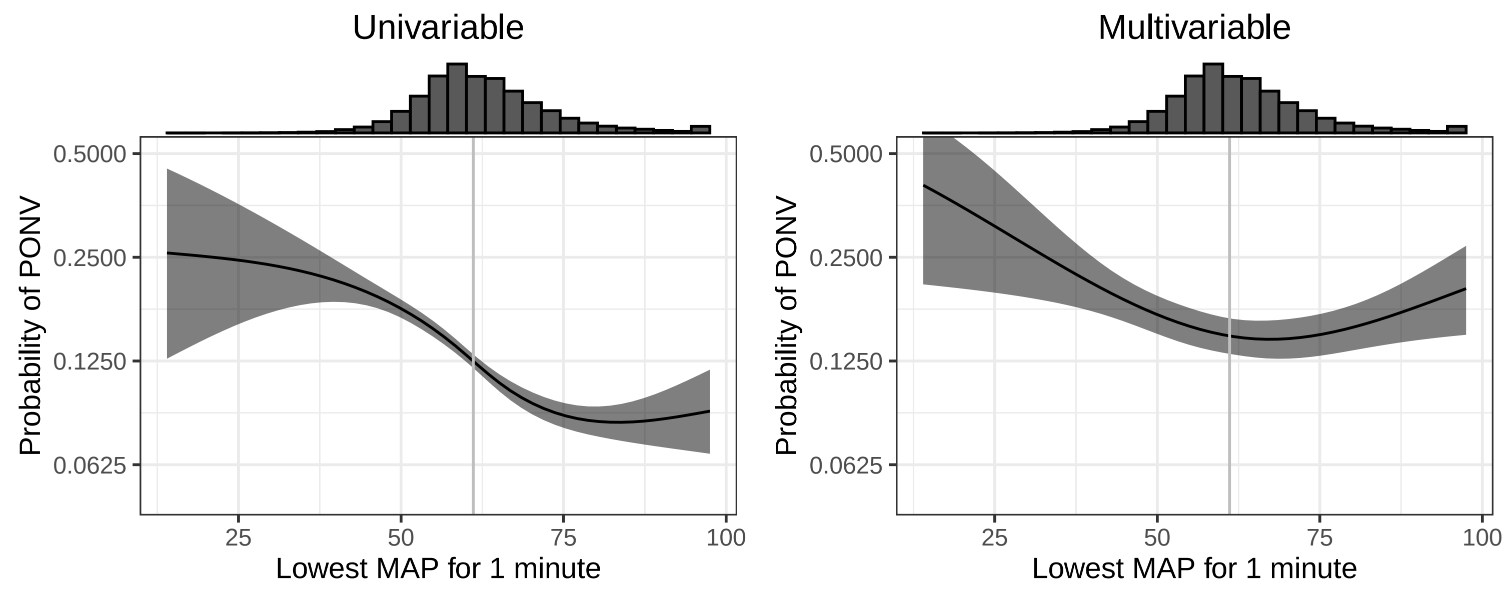

Univariable
Multivariable
Probability of PONV
0.5000
0.2500
0.1250
0.0625
25
50
75
100
Lowest MAP for 1 minute

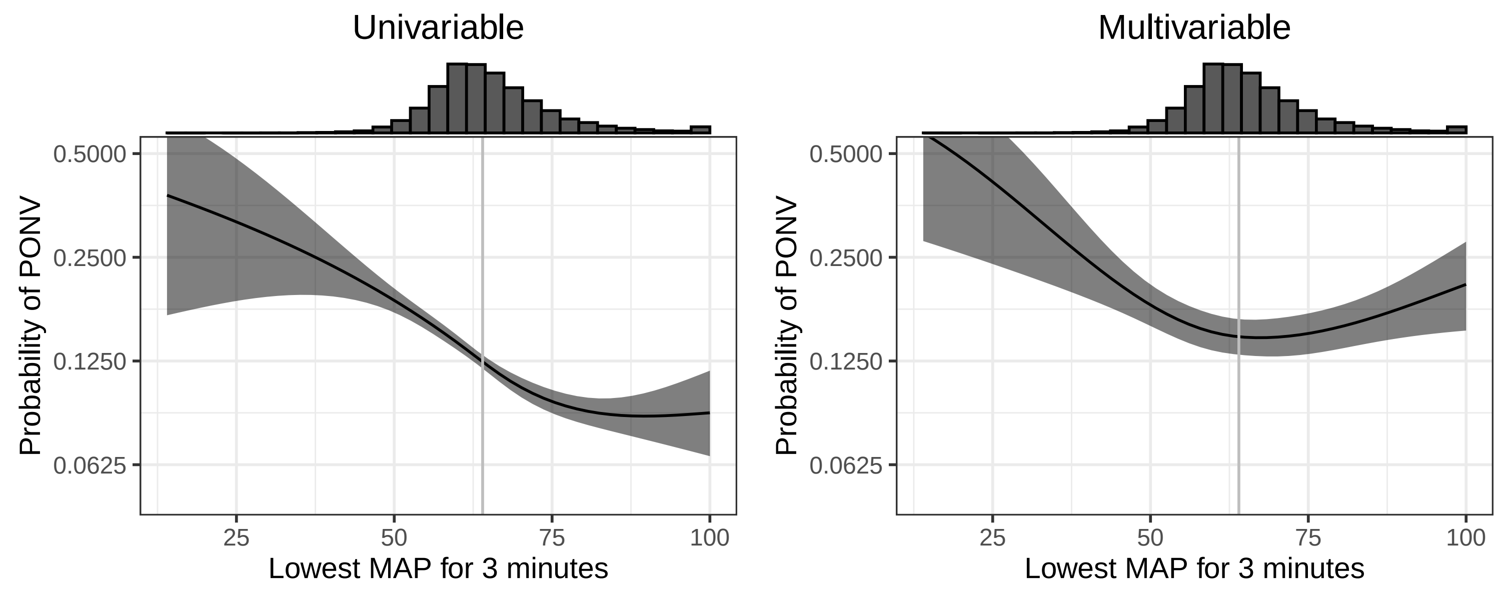

Univariable
Multivariable
Probability of PONV
0.5000
0.2500
0.1250
0.0625
25
50
75
100
Lowest MAP for 3 minutes

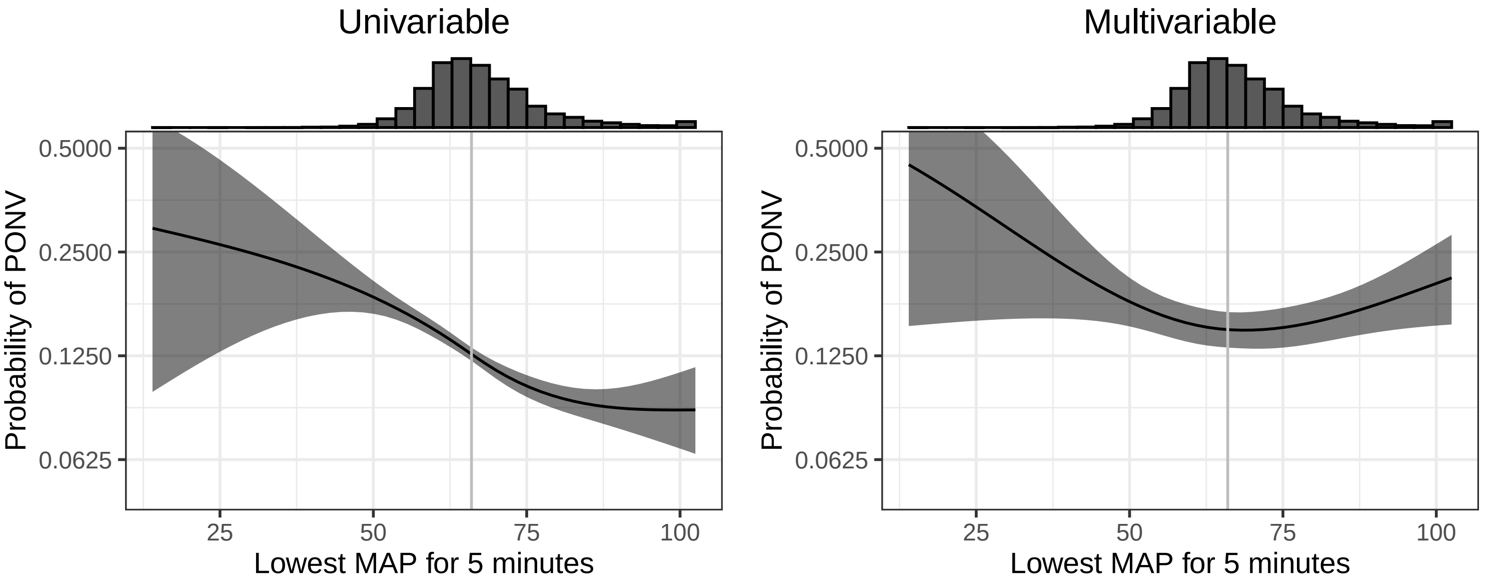

Univariable
Multivariable
Probability of PONV
0.5000
0.2500
0.1250
0.0625
25
50
75
100
Lowest MAP for 5 minutes

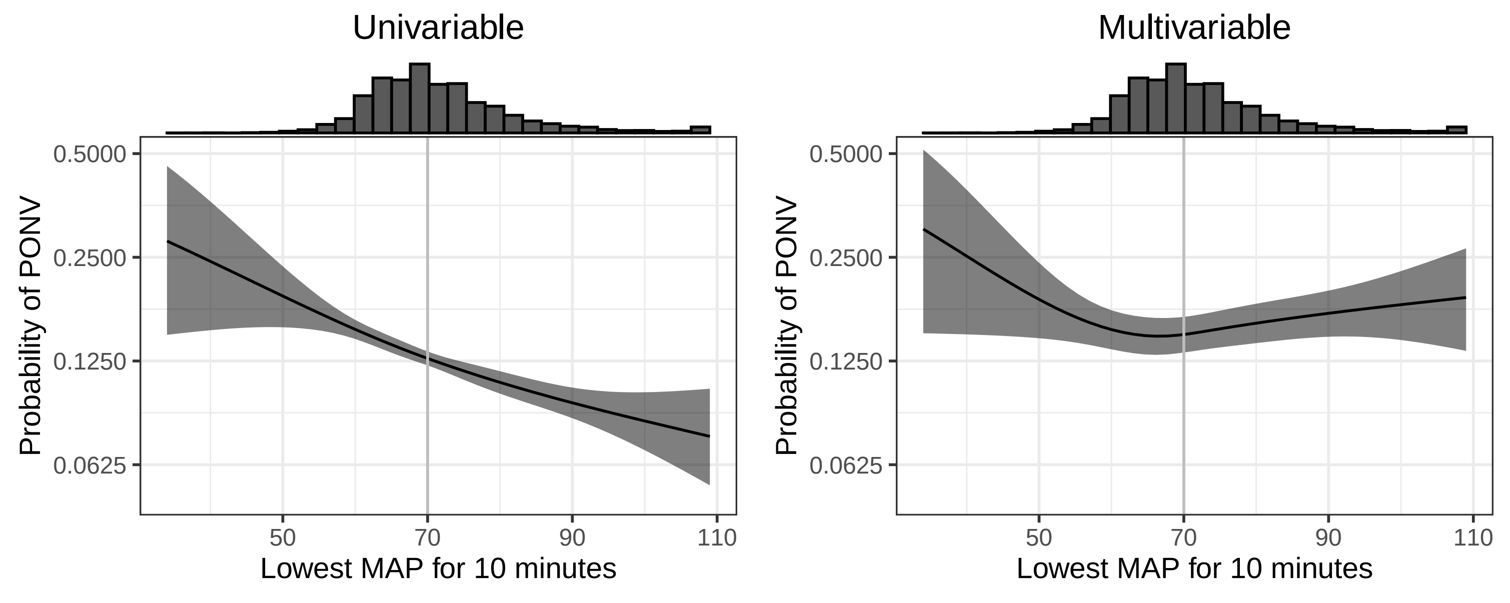
Univariable
Multivariable
0.5000
0.2500
0.1250
0.0625
Probability of PONV
50
70
90
110
Lowest MAP for 10 minutes

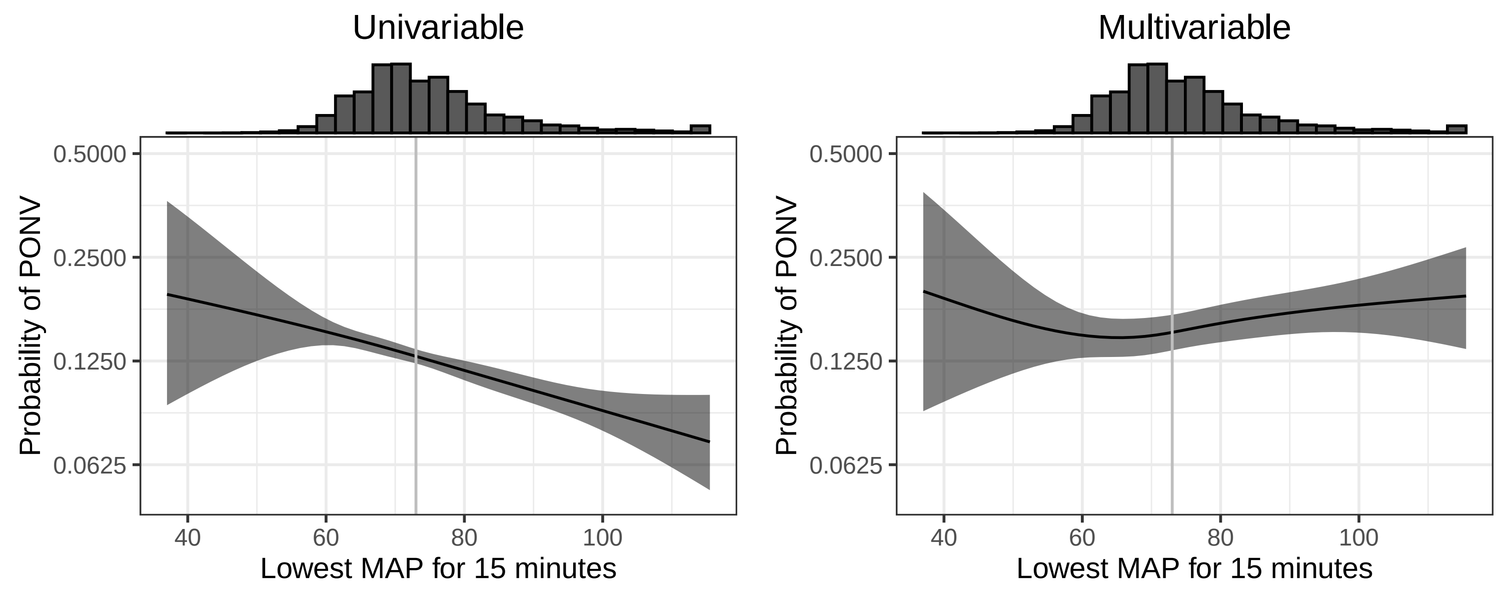

Univariable
Multivariable
0.5000
0.2500
0.1250
0.0625
Probability of PONV
40
60
80
100
Lowest MAP for 15 minutes

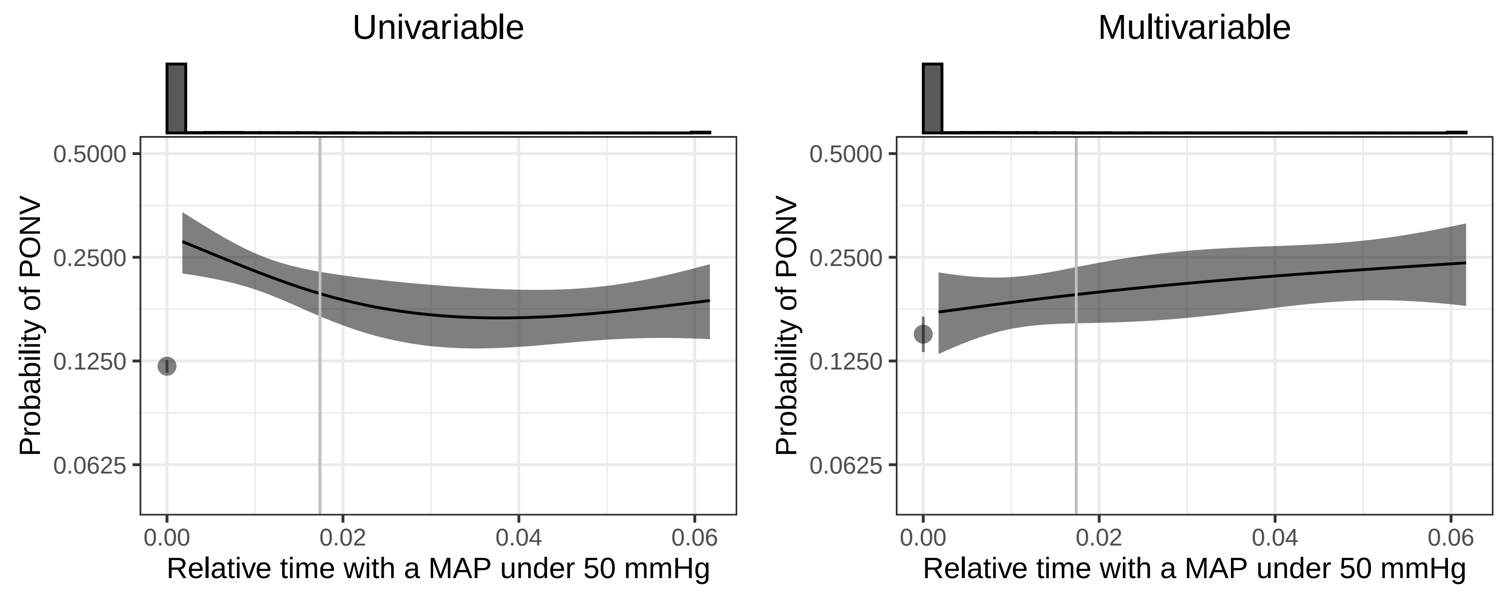

Univariable
Multivariable
0.5000
0.2500
0.1250
0.0625
Probability of PONV
0.00
0.02
0.04
0.06
Relative time with a MAP under 50 mmHg

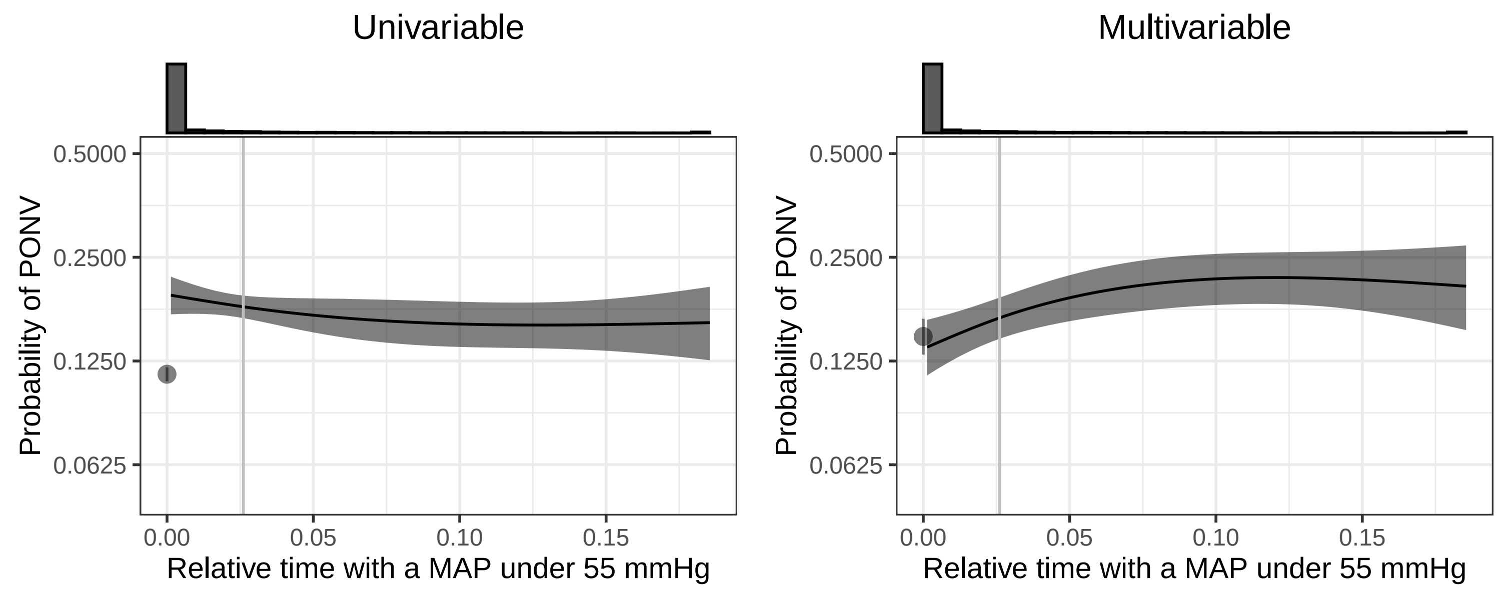

Univariable
Multivariable
Probability of PONV
0.5000
0.2500
0.1250
0.0625
0.00
0.05
0.10
0.15
Relative time with a MAP under 55 mmHg

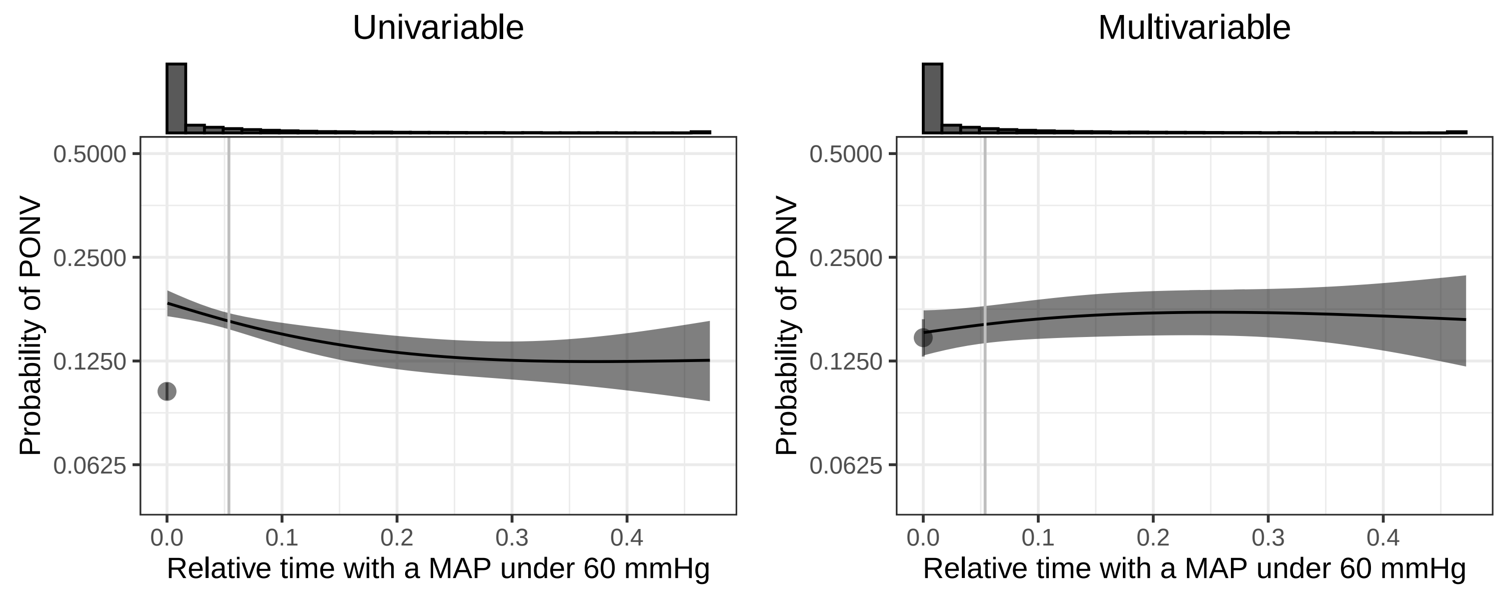

Univariable
Multivariable
Probability of PONV
0.5000
0.2500
0.1250
0.0625
0.0
0.1
0.2
0.3
0.4
Relative time with a MAP under 60 mmHg

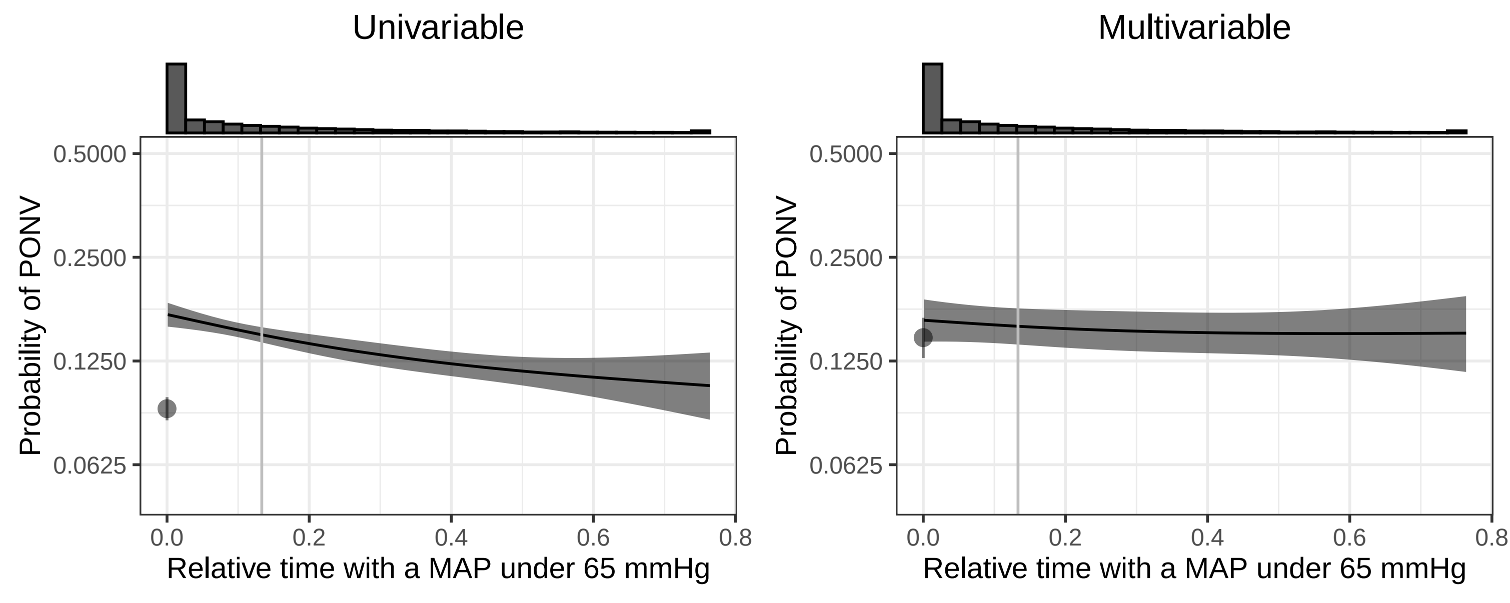
Univariable
Multivariable
0.5000
0.2500
0.1250
0.0625
Probability of PONV
0.0
0.2
0.4
0.6
0.8
Relative time with a MAP under 65 mmHg

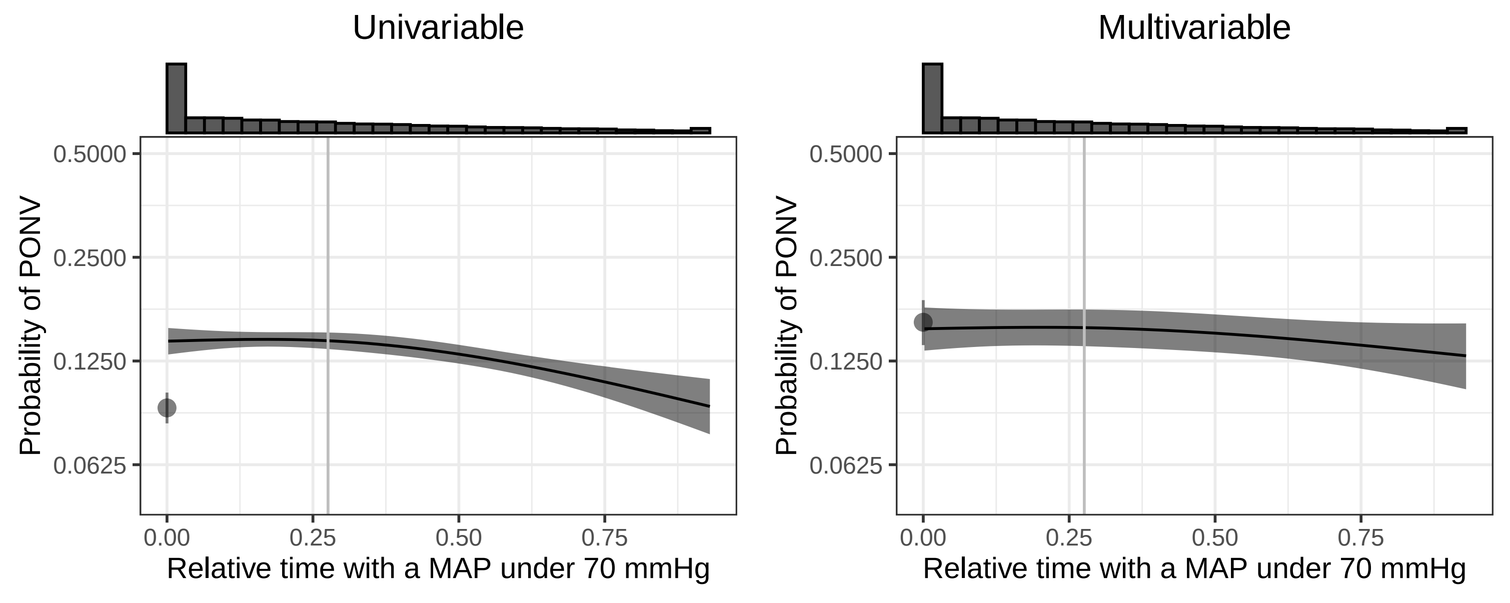

Univariable
Multivariable
Probability of PONV
0.5000
0.2500
0.1250
0.0625
0.00
0.25
0.50
0.75
Relative time with a MAP under 70 mmHg

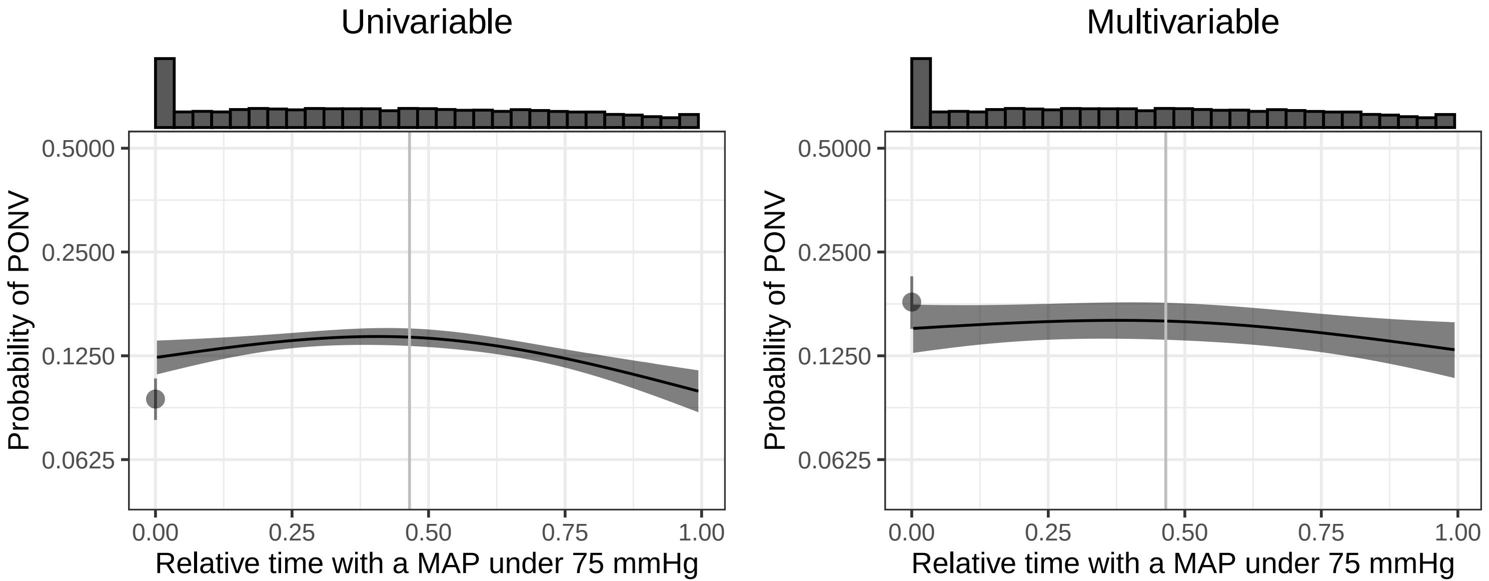

Univariable
Multivariable
Probability of PONV
0.5000
0.2500
0.1250
0.0625
0.00
0.25
0.50
0.75
1.00
Relative time with a MAP under 75 mmHg

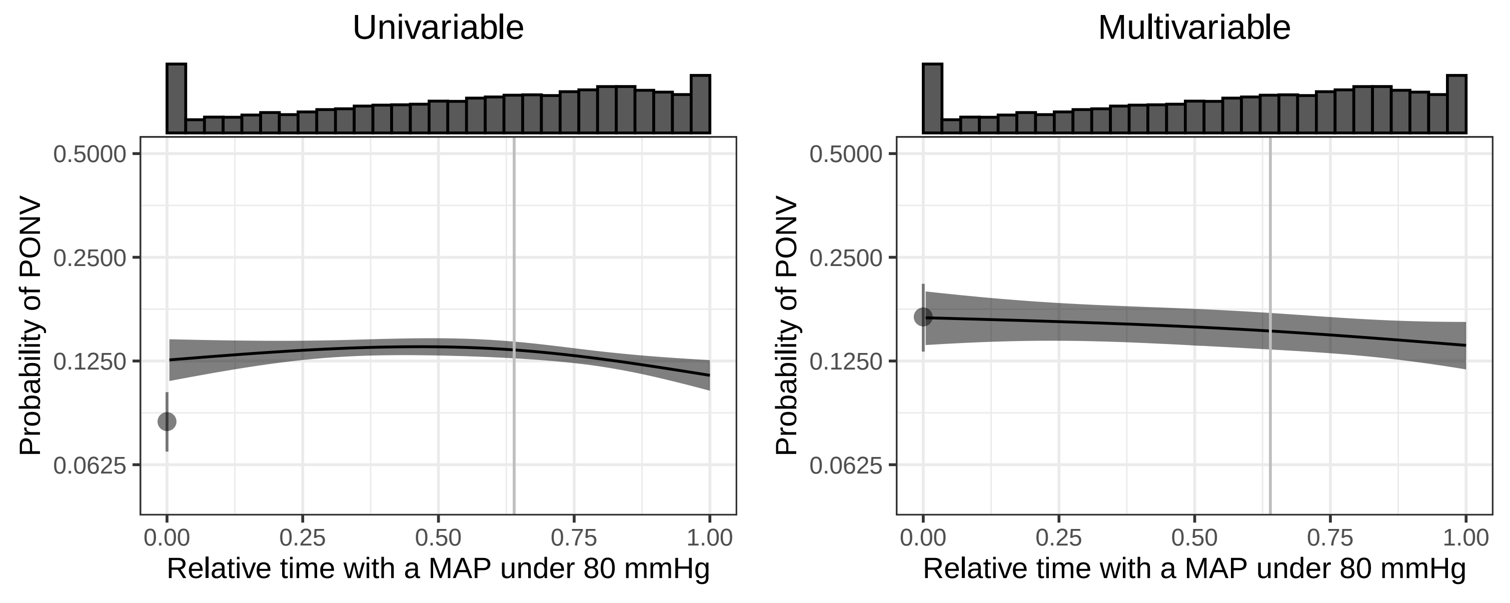
Univariable
Multivariable
Probability of PONV
0.5000
0.2500
0.1250
0.0625
0.00
0.25
0.50
0.75
1.00
Relative time with a MAP under 80 mmHg

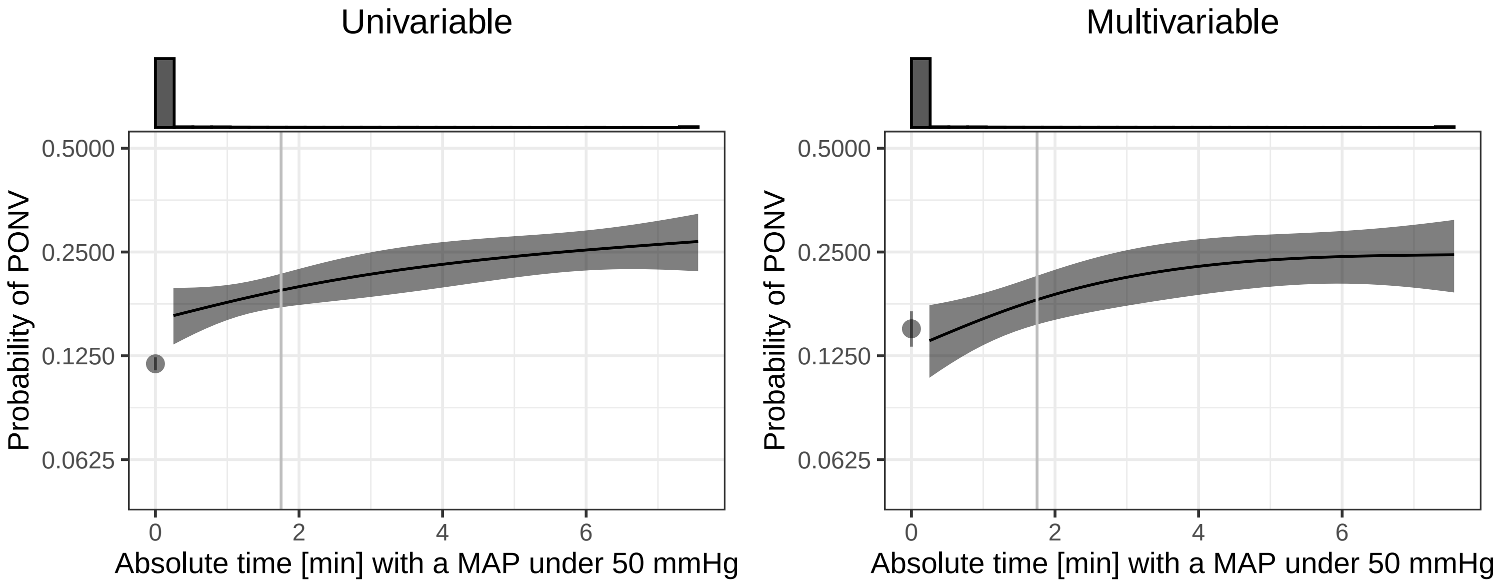
Univariable
Multivariable
0.5000
0.2500
0.1250
0.0625
Probability of PONV
0
2
4
6
Absolute time [min] with a MAP under 50 mmHg

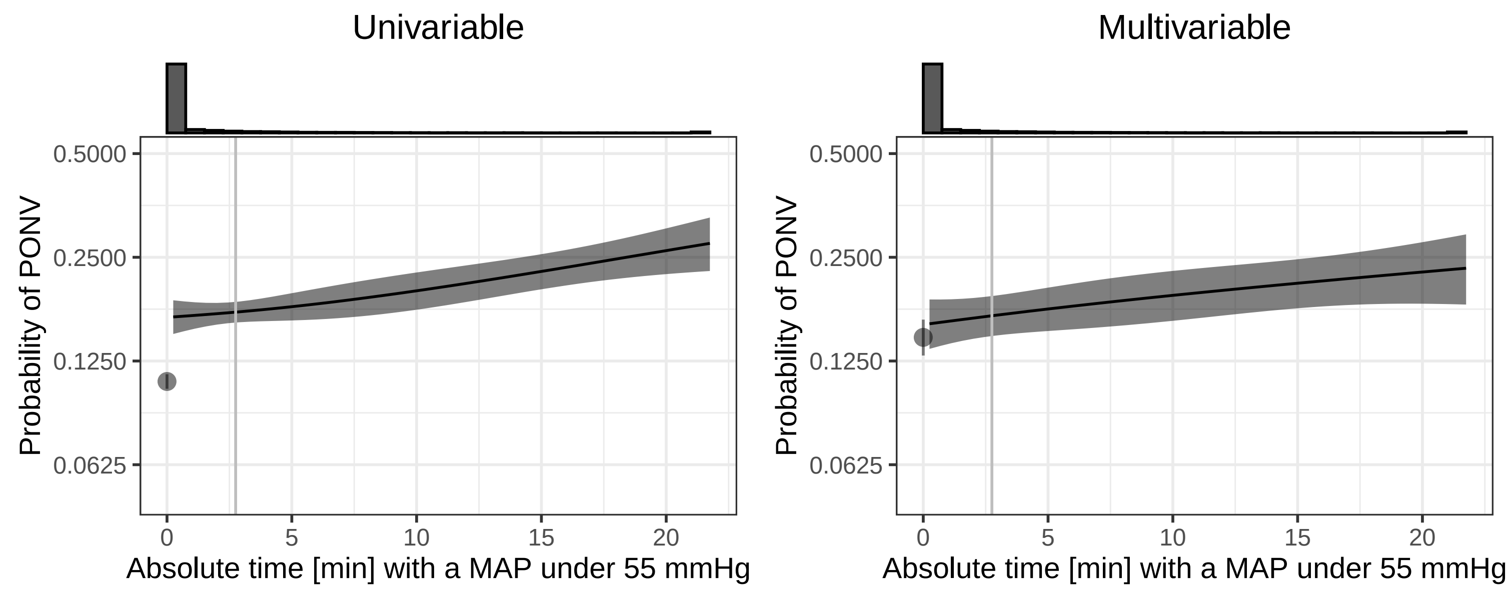
Univariable
Multivariable
Probability of PONV
0.5000
0.2500
0.1250
0.0625
0
5
10
15
20
Absolute time [min] with a MAP under 55 mmHg

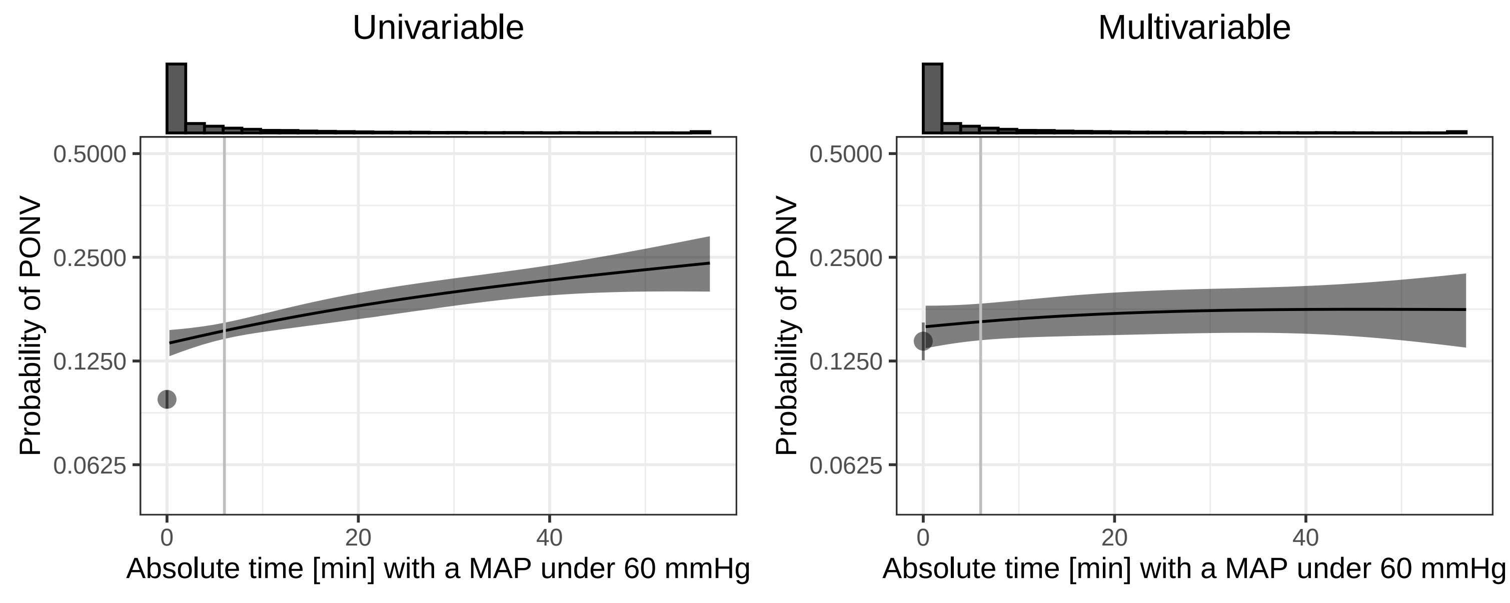

Univariable
Multivariable
0.5000
0.2500
0.1250
0.0625
Probability of PONV
0
20
40
Absolute time [min] with a MAP under 60 mmHg

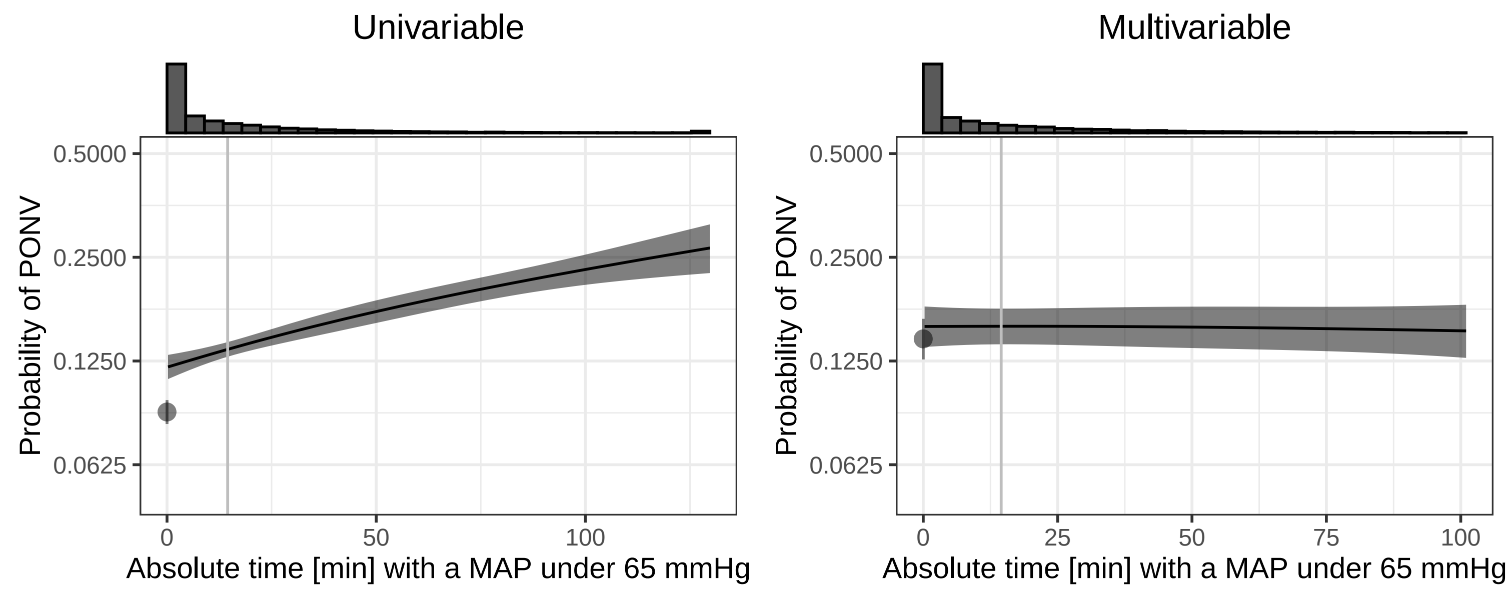

Univariable
Multivariable
Probability of PONV
0.5000
0.2500
0.1250
0.0625
0
50
100
25
75
Absolute time [min] with a MAP under 65 mmHg

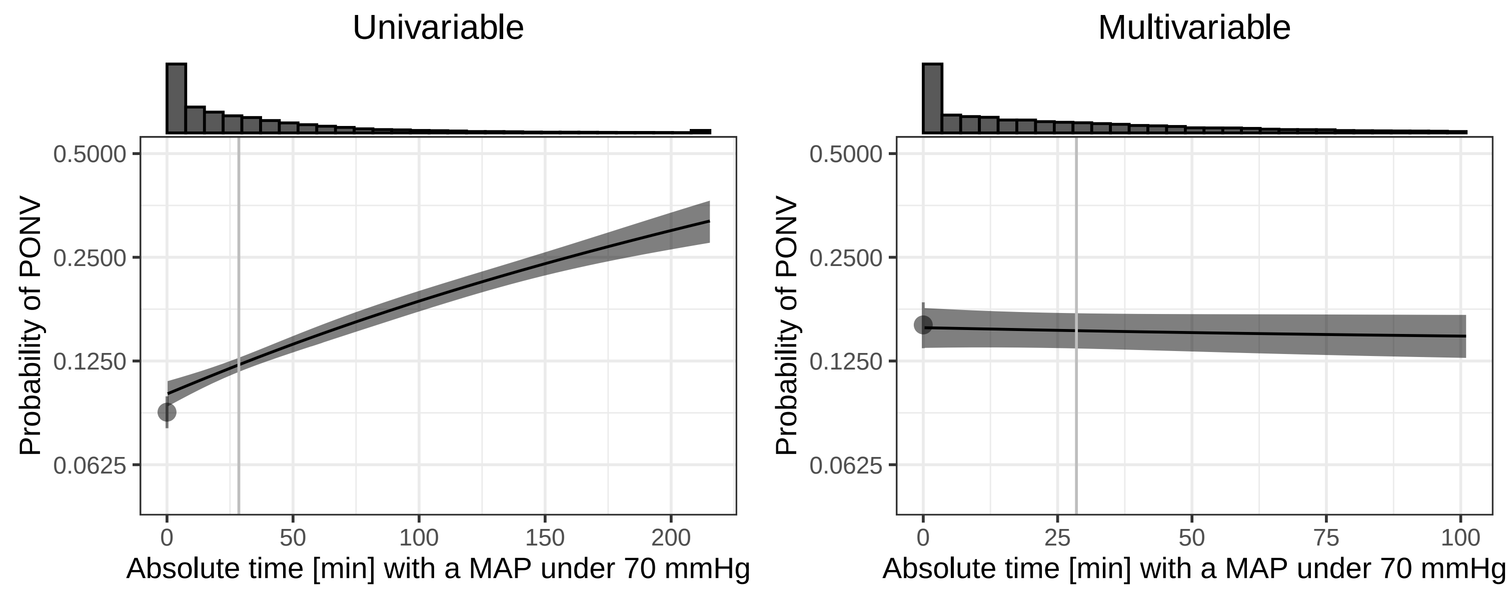

Univariable
Multivariable
Probability of PONV
0.5000
0.2500
0.1250
0.0625
0
50
100
150
200
25
75
Absolute time [min] with a MAP under 70 mmHg

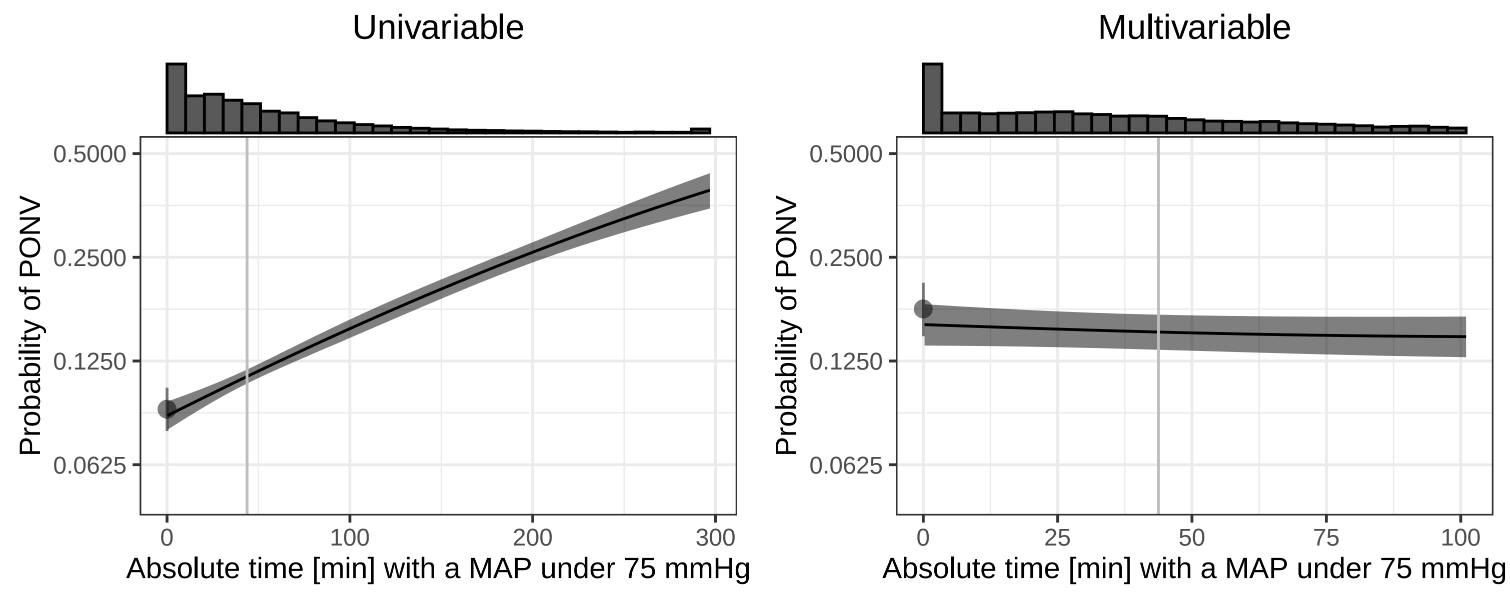

Univariable
Multivariable
Probability of PONV
0.5000
0.2500
0.1250
0.0625
0
100
200
300
25
50
75
Absolute time [min] with a MAP under 75 mmHg

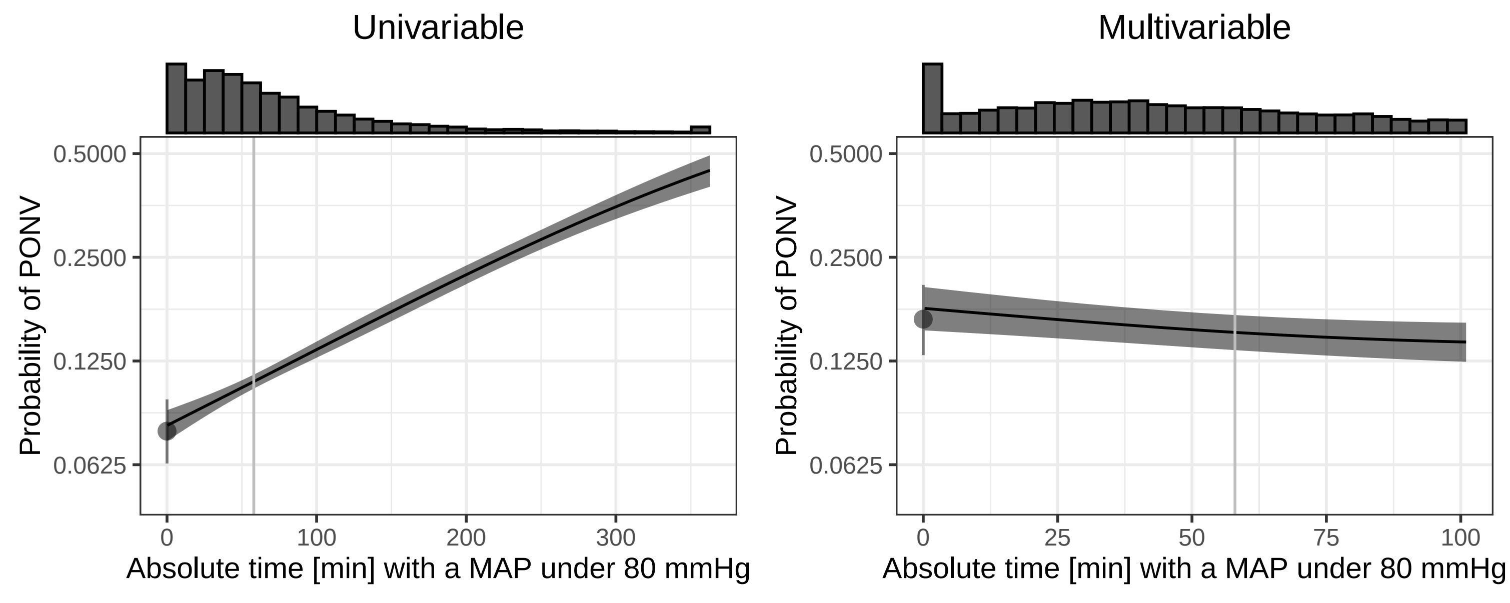

Univariable
Multivariable
Probability of PONV
0.5000
0.2500
0.1250
0.0625
0
100
200
300
25
50
75
Absolute time [min] with a MAP under 80 mmHg

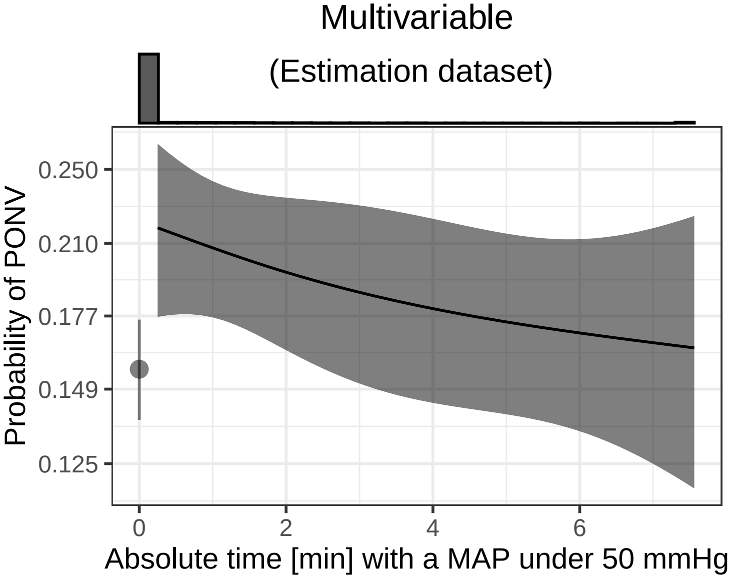

Multivariable
(Estimation dataset)
Probability of PONV
0.250
0.210
0.177
0.149
0.125
0
2
4
6
Absolute time [min] with a MAP under 50 mmHg
